# Oh my gut! Is the microbial origin of neurodegenerative diseases real?

**DOI:** 10.1128/iai.00437-22

**Published:** 2023-09-26

**Authors:** Alyssa Walker, Daniel M. Czyz

**Affiliations:** 1 Department of Microbiology and Cell Science, University of Florida, Gainesville, Florida, USA; University of California at Santa Cruz, Santa Cruz, California, USA

**Keywords:** proteostasis, human gut microbiota, Alzheimer's disease, Parkinson's disease, protein conformational diseases, neurodegenerative diseases, *Caenorhabditis elegans*

## Abstract

There is no cure or effective treatment for neurodegenerative protein conformational diseases (PCDs), such as Alzheimer’s or Parkinson’s diseases, mainly because the etiology of these diseases remains elusive. Recent data suggest that unique changes in the gut microbial composition are associated with these ailments; however, our current understanding of the bacterial role in the pathogenesis of PCDs is hindered by the complexity of the microbial communities associated with specific microbiomes, such as the gut, oral, or vaginal microbiota. The composition of these specific microbiomes is regarded as a unique fingerprint affected by factors such as infections, diet, lifestyle, and antibiotics. All of these factors also affect the severity of neurodegenerative diseases. The majority of studies that reveal microbial contribution are correlational, and various models, including worm, fly, and mouse, are being utilized to decipher the role of individual microbes that may affect disease onset and progression. Recent evidence from across model organisms and humans shows a positive correlation between the presence of gram-negative enteropathogenic bacteria and the pathogenesis of PCDs. While these correlational studies do not provide a mechanistic explanation, they do reveal contributing bacterial species and provide an important basis for further investigation. One of the lurking concerns related to the microbial contribution to PCDs is the increasing prevalence of antibiotic resistance and poor antibiotic stewardship, which ultimately select for proteotoxic bacteria, especially the gram-negative species that are known for intrinsic resistance. In this review, we summarize what is known about individual microbial contribution to PCDs and the potential impact of increasing antimicrobial resistance.

## BACTERIAL INFECTIONS, ANTIBIOTICS, AND PROTEIN CONFORMATIONAL DISEASES

The early broad therapeutic applications of antimicrobials, mainly heavy metals and other toxic chemicals, were influenced by the Germ Theory of Disease during the early 19th century (1). Interestingly, the initial diagnoses of neurodegenerative protein conformational diseases (PCDs), such as Alzheimer’s disease (AD), Parkinson’s disease (PD), Huntington’s disease (HD), or amyotrophic lateral sclerosis (ALS) coincide with the same period (2 – 5), suggesting that, in addition to heavy metal toxicity, antimicrobials could have contributed to the pathogenesis of these diseases. Although, the initial diagnoses of PCDs could have been a result of the rapid population expansion or increase in medical practice that led to better diagnosis. However, what is more striking is that the incidence of each of these diseases began to rapidly increase following the discovery and commercialization of antibiotics, especially after the second half of the 20th century post the “Golden Age” of antibiotics (6). There is stronger evidence suggesting that microbes contribute to the pathogenesis of PCDs, but could antimicrobials also contribute to these diseases by eradicating the protective microbiota? If so, do antibiotics eliminate protective microbes and enrich for detrimental bacteria? How will the growing antimicrobial resistance (AMR) among pathogenic bacteria affect the incidence of PCDs? In this review, we will attempt to address these intriguing questions by summarizing the current literature.

The first suggestion that bacteria may be affecting the pathogenesis of PCDs, or more specifically Parkinson’s disease, was postulated by Braak (7); however, the evidence of the microbial origin of PCDs can be seen throughout the literature much earlier. For example, prior to the discovery of antibiotics, syphilis, a sexually transmitted infection caused by *Treponema pallidum*, a spirochaete bacterium, was the leading cause of paralytic dementia, a neurological condition that manifests in rapid cognitive decline, which accounted for a significant rise in asylum admissions (8, 9). Spirochetal infections were later linked to AD (10, 11). One of the first successful treatments at the beginning of the 20th century was possible due to the discovery and commercialization of Salvarsan, an arsenic-based antimicrobial, followed by the discovery of penicillin, which provided effective remedies and consequently eliminated neurological complications associated with *T. pallidium* infection (12, 13). Further evidence of microbial contribution to neurodegenerative diseases was observed in Japanese leprosy patients treated with dapsone, an antimicrobial sulfa drug. The patients who were administered the drug were more than twice less likely to develop dementia (14). It remains unknown whether the protective property of dapsone was the result of any off-target effects or whether the drug indirectly affected the disease pathogenicity by targeting *Mycobacterium leprae*. Another study has linked mycoplasma presence to ALS. Nicolson et al. found that 83% (30/36) of ALS patients had mycoplasma detected in blood cultures compared to only 2.8% in the control group (2/70) (15). Mycoplasma pneumonia has also been linked to acute parkinsonism (16). More recently, antibiotics have been shown to alleviate disease phenotypes in PCD patients suffering from chronic infection. For example, 88% of AD patients were diagnosed with *Helicobacter pylori* infection compared to 46.7% of controls, and eradication of the pathogen was associated with decreased disease progression in both a clinical trial and a population-based study (17 – 19). *H. pylori* eradication with an antibiotic cocktail consisting of omeprazole, clarithromycin, and amoxicillin improved AD-associated symptoms, enhancing cognitive and functional skills (18). Furthermore, a population-based study revealed that eliminating *H. pylori* decreases dementia progression (19). Enteric infections caused by *H. pylori* have been associated with increased intestinal permeability (20), and Parkinson’s disease pathogenicity (21, 22). An increase in intestinal permeability can lead to the dissemination of the intestinal content to other parts of the body. In fact, bacteria and their products have been found in the brains of people with PCDs, including AD (23 – 25). Other bacteria, such as group B *Streptococcus* (GBS), are part of the commensal microbiota that asymptomatically colonize various sites of the human microbiome (26). GBS is known to disrupt intercellular junctions of intestinal epithelial cells (27), which likely contributes to changes in intestinal permeability, leading to a displacement of bacteria and possibly other members of the gut microbiota. Interestingly, acute parkinsonism has been observed in post-streptococcal infections in young individuals (28 – 30). The link between intestinal permeability and brain disorders is evident (31), as elevated levels of circulating lipopolysaccharide (LPS), a bacterial endotoxin that contributes to PCD pathogenicity (32, 33), have been detected in elderly patients, patients with chronic infection (34), and in AD and ALS patients (23, 35). LPS has also been shown to contribute to the pathology of PD in mouse models (36, 37). The collective evidence suggests that antibiotics provide a level of protection against neurodegenerative diseases, but likely in the context of the infection.

Although antibiotics seem to have an inhibitory effect on the pathogenicity of PCDs, the benefits are not evident in the absence of infection. Certain antibiotics, for example, tetracyclines, were promising therapeutic candidates as they were found to directly interact with different protein amyloids and interfere with their aggregation *in vitro* (38 – 41); however, they completely failed to provide any protection in clinical trials (42). An increasing body of evidence supports the proteotoxic role of antibiotics on the pathogenicity of neurodegenerative diseases. For instance, antibiotics were found to increase the risk of PCDs in two nationwide case studies (43, 44). Sun et al. analyzed antibiotic use among 2,484 Swedish ALS patients and found an increased risk associated with all antibiotics, especially β-lactamase-sensitive penicillin (44). While these results suggest that the disruption of the protective microbiota might contribute to the disease pathogenesis, other studies showed a protective effect of penicillin (45). Such discrepancy in the influence of antibiotics on ALS pathogenicity is likely attributed to two factors: disruption of the protective microbiota and elimination of proteotoxic bacteria. As such, in the absence of any underlying infection, penicillin administration would be either detrimental, as observed by Sun et al., or have no effect on ALS pathogenicity—a result that was observed in a small clinical trial (46). The detrimental effect of antibiotics on ALS was also seen in patients treated with a tetracycline-class antibiotic, minocycline, which accelerated disease progression and increased mortality (47). Tetracyclines are known to inhibit bacterial growth by reversibly binding to the prokaryotic ribosome and ultimately blocking translation (48); however, recent data suggest that tetracyclines can also interact with the eukaryotic ribosome (49), which could affect eukaryotic translation rate and ultimately alleviate the expression of destabilized aggregation-prone proteins that are most sensitive to proteotoxicity. While numerous studies have demonstrated the neuroprotective effect of minocycline in animal models, little evidence exists to support its efficacy in humans (50, 51). Minocycline’s lack of efficacy in humans may not be surprising given a 100% failure rate of potential therapeutics that were initially tested in around 170 transgenic murine models of AD (52). Such discrepancy between tetracycline efficacy *in vitro* and *in vivo* animal models vs humans may be mediated by the differences in human and rodent gut microbiota, which only share a mere 10% of bacterial species (53). In agreement with the detrimental effect of antibiotics in the absence of infection, a large Finnish study analyzed the effect of antibiotic usage on PD risk. The analysis of nearly 14,000 PD patients revealed an elevated risk of developing the disease after exposure to different antimicrobials, including macrolides, lincosamides, tetracyclines, sulfonamide, and trimethoprim (43). In a recent study that included 14,000 women, Mehta et al. noted a detectable cognitive decline 7 years post mild antibiotic use (54). Furthermore, a recent analysis of early- and mid-life antibiotic usage within the Swedish population revealed a significant association with AD and PD (55). The detrimental effect of antibiotics is further supported by another study that linked the duration of antibiotic treatment with increased risk for dementia in a cohort of 313,161 participants (56). Collectively, these findings indicate that the protective effect of antibiotics is likely associated with the eradication of proteotoxic bacteria that contribute to the pathogenesis of PCDs, and general antibiotic use could increase the risk of PCDs later in life, possibly by altering the protective microbiome.

Antibiotics might increase the risk for neurodegenerative diseases by depleting bacteria that confer neuroprotection to the host. For example, clinical data reveal that the abundance of bacteria from the *Prevotella* genus negatively correlates with the pathogenicity and prevalence of different PCDs, including ALS (57), PD (58 – 66), and a mouse AD model (67). A decreased abundance of *Prevotella* spp. is also associated with constipation, a condition that precedes motor symptoms in PD patients by up to 20 years (46, 68). These results suggest that changes in the gut microbiota are the cause and not the effect of the disease. According to the Global Biodiversity Information Facility, there are 61 species of *Prevotella* (69), but only about 20 are associated with human diseases (70). As such, reporting results at the genus level hinders our true understanding of bacterial contribution. While the mechanisms underlying the neuroprotective role of *Prevotella* spp. remain unknown, recent work revealed that specific species (i.e., *disiens* and *corporis*) decrease toxic polyglutamine (polyQ) aggregation in *Caenorhabditis elegans* (71, 72). Studies in pigs and rats have shown that antibiotic use reduces the relative abundance of *Prevotella* spp. (73, 74), suggesting that a similar effect can be expected in humans, which may support the link between antibiotics and neurodegenerative diseases (43, 44). Ampicillin was shown to significantly alter the composition of the microbiota in rats and was associated with physiological and psychological changes, including two hallmarks of AD: memory defects and elevated glucocorticoid levels (75). The aforementioned issues, as well as gut dysbiosis, were rescued with probiotic supplementation of *Lactobacillus fermentum*, a commensal strain that is known for its potent antioxidative property (76). Several studies have demonstrated the beneficial effect of *Lactobacillus* spp. For example, a probiotic cocktail that included *L. fermentum* and *L. plantarum* delayed the onset and progression of AD in *Drosophila* (77), whereas another probiotic cocktail containing *Lactobacillus* spp. alleviated PD symptoms in mice (78) and reduced AD symptoms in rats (79). What is interesting to note is that while 100% of therapeutics that effectively treated AD in animal models failed in human clinical trials, this is not the case with bacteria-mediated approaches. For example, a double-blind, randomized study demonstrated that a probiotic cocktail containing *Lactobacillus* spp., including *fermentum*, improved cognition in people with AD after 12 weeks of supplementation (80). Together, these results accentuate the risk of antibiotic-induced gut dysbiosis, particularly in the context of neurodegenerative PCDs.

Our recent work revealed that host proteostasis is affected by changes in the bacterial proteome. We showed that colonization of the *C. elegans* intestine with bacteria that have an increased abundance of protein aggregates leads to disruption of the host proteostasis (72). Walker et al. demonstrated that gentamicin, an antibiotic from the aminoglycoside class known to kill bacteria by inducing mistranslation and protein misfolding (81), not only increased the abundance of bacteria-derived protein aggregates (BDPAs) but also enhanced polyQ aggregation in *C. elegans* colonized with bacteria exposed to this antibiotic (72). Since antibiotics are specifically targeting bacteria, these results suggest that they may also indirectly affect the stability of the host proteome. In fact, antibiotics can induce uncharacterized BDPAs that play a physiological role in bacterial virulence and persistence (82). Together, these results raise the concern that antibiotics may not be as inert on the host as is generally accepted, and they can contribute to the pathogenesis of PCDs by affecting the composition of the human microbiome, as well as enhancing BDPAs.

**Fig 1:**
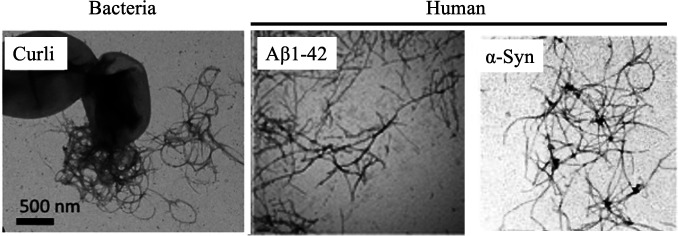
Bacterial and human protein aggregates. (Left) Bacterial curli (reproduced from reference (86) with permission). (Middle) Human Aβ1-42 (reproduced from reference (92) with permission). (Right) Human α-synuclein (reproduced from reference (93) with permission).

While it is estimated that the human gut harbors at least as many bacteria as there are cells in the human body, the number of bacterial genes far exceeds that of humans by a staggering projection of 100- or more-fold (83, 84). Therefore, it should not be surprising that there are bacterial genes that affect host proteostasis and ultimately contribute to the pathogenesis of PCDs. Gram-negative bacteria, mainly from the *Enterobacteriaceae* family (85), produce curli which are functional amyloids that aggregate to form an extracellular matrix that closely resembles aggregates associated with PCDs, such as AD or PD (Fig. 1). These bacterial functional amyloids play a role in cell adhesion, biofilm formation, and antibiotic resistance, and recent studies suggest that these proteins may also be implicated in the pathogenesis of PCDs (86 – 91).

For example, FapC, the amyloid produced by *Pseudomonas,* affected *in vitro* aggregation of α-synuclein and amyloid beta (Aβ), and enhanced AD pathogenesis in zebrafish (94, 95). Interestingly, about 80% of cystic fibrosis (CF) patients are colonized by *Pseudomonas aeruginosa* by the age of 18 (96). Despite CF being a non-neurodegenerative PCD, recent studies show that CF patients exhibit cognitive dysfunction and brain tissue changes (97, 98). The life expectancy of those affected with CF ranges between 30 and 40 years, which is about 20–30 years less than the average diagnosis age of PCDs such as PD or AD (99, 100). Therefore, it is possible that this age difference is the reason for no obvious correlation between CF and neurodegenerative PCDs; however, it is striking that CF patients develop secondary amyloidosis due to aggregation of serum amyloid A protein (SAA) (101). Interestingly, the levels of SAA were shown to correlate with *P. aeruginosa* presence in the lungs of CF patients and were attenuated with antibiotics, further supporting our hypothesis that bacteria, in this case, *P. aeruginosa*, contribute to the disruption of host proteostasis (102).

Additional indirect evidence suggesting that antibiotics are linked to neurodegenerative diseases comes from their usage within the general population, where women are more commonly prescribed antibiotics, and in some studies, the difference accounts for nearly twice as many prescriptions when compared to men (103, 104). Surprisingly, the lifetime risk for developing Alzheimer’s dementia at the ages 45 and 65 is nearly twofold higher for women compared to men (105, 106). These results support the finding by Mehta et al., who reported that antibiotic use in women during midlife is associated with decreased cognition 7 years later (54). Furthermore, women have a lower abundance of *Prevotella* spp. in their gut, which, given the suspected neuroprotective property of these bacteria, may contribute to the difference in the risk for AD between women and men (107 – 109). Based on the correlation between *Prevotella* abundance and antibiotic use in animals (73, 74), the increased use of antibiotics may be linked to a lower abundance of *Prevotella* and an increased risk for AD and dementia. Other evidence linking antibiotics with AD is based on the correlation between AD prevalence and antibiotic consumption. Interestingly, based on 2018 surveillance data, Italy and Greece had the highest dementia prevalence among all European countries (110). In 2010, these two countries were also one of the largest consumers of antibiotics (111), and in 2010, Italy was also the largest user of antibiotics in livestock (112). These data reveal a surprising correlation between global antibiotic consumption and dementia and may support previous reports that link antibiotic usage to elevated risk for several neurodegenerative diseases, including AD and dementia (43, 44, 47, 56). In addition to global antibiotic consumption and sex-specificity, the recent coronavirus disease 2019 (COVID-19) pandemic has increased the risk for dementia and dementia-related deaths, likely due to social isolation (113), reduced accessibility to social activities due to lockdowns and social distancing (114, 115), and most importantly, the overuse of antibiotics (116). Moreover, it was recently estimated that COVID-19 increased the risk for AD twofold, and AD-related deaths increased by 16% (117, 118). While it is not clear whether the virus directly affects the pathology of AD or whether the effect comes from the abovementioned consequences of the pandemic, the evidence points toward the inflammatory effect of the virus, secondary infections, and poor antibiotic stewardship—all of which are known to contribute to the pathogenesis of PCDs. A survey of 10,403 pneumonia patients revealed that 3% of the affected individuals who contracted COVID-19-related pneumonia developed new-onset dementia, an incidence that is much higher than in non-COVID-19 patients (119). Pneumonia was previously linked to PCDs in critically ill patients (120). In one study, it was noted that hospitalization with COVID-19 was associated with a cognitive decline, where the incidence reached over 26% in patients with severe COVID-19 (121). A recent pre-print also reported the presence of amyloid deposits in the brains of patients with severe COVID-19 (122). It is important to note that severe COVID-19 patients are subjected to empiric antibiotic treatment (123). Furthermore, in a Danish population-based study, Zarifkar et al. found that COVID-19 increased the frequency of AD and PD by 3.4 and 2.2 times, respectively (124). The study found that most neurological disorders were no more frequent after COVID-19 than after other respiratory infections, which suggests that the detrimental effect could be due to antibiotic treatment or general inflammation.

While the link between COVID-19 and AD could be explained by the disrupted antibiotic stewardship and underlying secondary bacterial infections, the potential role of other viral infections in AD pathogenesis has also been established, though the viral contribution to PCDs is another extensive topic that is outside the scope of this review. Nonetheless, it is important to emphasize that in addition to coronavirus other viruses including herpes simplex, human cytomegalovirus, Epstein-Barr virus, hepatitis C, picornavirus, Borna disease virus, and influenza were associated with cognitive decline, and in some instances their presence correlated with AD and other PCDs (125 – 127). Unlike common extracellular bacteria, viruses are obligate intracellular parasites and as a result, hijack the cellular protein homeostasis machinery, often sequestering heat shock proteins away from their endogenous roles (128). As such, it is not surprising that many of the aforementioned viruses activate the heat shock response and could potentially lead to misfolding and aggregation of any destabilized disease-associated host proteins (129). Additionally, viruses are also known to affect neurons leading to their degeneration (130, 131).

Antibiotics directly target bacteria and can rapidly alter the composition of the gut microbiota, but they are not the only factor that influences the microbial balance (132). Factors such as alcohol consumption (133, 134), smoking (135), physical activity (136), and diet are known to affect the gut microbiota (137). Lourida et al. looked at the association between these factors and dementia in a cohort of nearly 200,000 participants. Not surprisingly, a healthy lifestyle associated with moderate alcohol consumption, no smoking, regular physical activity, and a healthy diet resulted in a lower risk of developing dementia (138).

In summary, numerous bacterial infections have been associated with the pathogenesis of neurodegenerative PCDs, and it does not seem that specific bacteria are linked to any particular PCD, but rather disrupt host proteostasis in general and lead to toxic protein aggregation that manifests in disease pathogenesis. Infection prevention and stricter antibiotic stewardship programs at any age need to be implemented in order to decrease the prevalence of PCDs.

## ASYMPTOMATIC COLONIZATION, ANTIMICROBIAL RESISTANCE, AND PROTEIN CONFORMATIONAL DISEASES

Antibiotics are essential medicines known as the wonder drugs, but due to increasing AMR, their efficacy is slowly fading. In fact, AMR is emerging as one of the largest global healthcare problems that is already associated with an estimated 4.95 million deaths annually (139). The number of deaths is projected to increase 10-fold by 2050 if no immediate actions are taken (140). AMR is a dire crisis that likely affects the prevalence of PCDs, as proposed in this review based on the current interpretation of the literature that sits at the interface of microbiology and neuroscience. Many of the bacterial species, especially the gram-negative, which are associated with PCDs are known for intrinsic and acquired antibiotic resistance. Often, these resistant species are linked to PCDs and are known to asymptomatically colonize the human gut. Administration of antibiotics eliminates the protective microbiota but enriches for the abundance of the resistant strains. Recent studies show that even a short duration of antibiotic treatment will select for resistant genes within the gut microbiome (141). An increase in the abundance of such asymptomatic colonizers driven by antibiotics can silently contribute to the pathogenesis of PCDs years before the onset of any symptoms. For instance, it was shown that antibiotic treatment could enrich for *P. aeruginosa in vitro* as well as *Klebsiella pneumoniae* and *Proteus mirabilis* in mice (142, 143). An increased presence of *P. aeruginosa* and *K. pneumoniae* in the gut has been associated with PCDs, and multidrug-resistant (MDR) strains of these bacteria are commonly found across hospitals and community (144 – 146). *P. mirabilis* is an opportunistic pathogen that is otherwise considered commensal, and its abundance was fivefold higher in septic PD patients compared to non-PD controls (147, 148). Furthermore, oral administration of these bacteria induced α-synuclein aggregation in mouse brains and enhanced PD-like symptoms (149) (Table 1 summarizes all bacteria-animal models). Interestingly, all three of the aforementioned pathogens were shown to increase toxic polyQ aggregation and the associated toxicity upon colonization of the *C. elegans* intestine (71).

**TABLE 1:** Summary of strain-specific effects on animal models of neurodegenerative diseases^*f*^

Bacteria	Model	Protein	Disease	Finding	Source	
*Acinetobacter baumannii*	Worm	polyQ	PolyQ^*b*^	Disrupted protein folding environment, which led to toxic polyQ aggregation.	(71)	
*Acinetobacter baylyi*	Worm	polyQ	PolyQ^*b*^	Disrupted protein folding environment, which led to toxic polyQ aggregation.	(71)	
*Agathobaculum butyriciproducens*	Mouse	N/A	PD	Positive effects on PD-related phenotypes.	(150)	
*Agathobaculum butyriciproducens*	Mouse	APP/PS1	AD	Alleviated cognitive defects in LPS-treated wild-type mice and decreased amyloid plaques in APP/PS1 mice.	(151)	
*Akkermansia muciniphila*	Mouse	APP/PS1	AD	Ameliorated cognitive defects and reduced Aβ plaques in the brain.	(152)	
*Akkermansia muciniphila*	Rat	Aβ	AD	Reduced Aβ deposits and alleviated cognitive impairment.	(153)	
*Bacillus subtilis*	Worm	α-synuclein	PD	Inhibited α-synuclein aggregation and cleared preformed aggregates.	(154)	
*Bacillus subtilis*	Worm	Aβ	AD	Delayed aging and neuronal deterioration in wild-type worms. In Aβ models, the bacterium alleviated motility defects and extended lifespan.	(155)	
*Bifidobacterium bifidum^c^*	Mouse	MitoPark^*a*^	PD	Probiotic cocktail had neuroprotective effects on dopaminergic neurons and counteracted motor impairments.	(78)	
*Bifidobacterium bifidum*	Mouse	APP/PS1 (5xFAD)	AD	Decreased cognitive function and AD pathology.	(156)	
*Bifidobacterium breve*	Mouse	Aβ	AD	Prevented cognitive impairments that were otherwise induced by intracerebroventricularly administered Aβ.	(157)	
*Bifidobacterium infantis^d^*	Rat	Aβ	AD	Improved memory and decreased Aβ plaques in rats that received hippocampal injections of Aβ.	(158)	
*Bifidobacterium longum^e^*	Fly	APP-BACE1	AD	Probiotic cocktail reduced the formation of Aβ plaques over time.	(77)	
*Bifidobacterium longum^c^*	Mouse	MitoPark^*a*^	PD	Neuroprotective effects on dopaminergic neurons and counteracted motor impairments.	(78)	
*Bifidobacterium longum*	Mouse	APP/PS1	AD	Alleviated cognitive decline and suppressed hippocampal accumulation of Aβ plaques.	(159)	
*Bifidobacterium longum*	Mouse	APP/PS1 (5xFAD)	AD	Decreased cognitive function and AD pathology.	(156)	
*Chlamydia pneumoniae*	Mouse	N/A	AD	Induced Aβ deposition in brain such that deposit density, size, and number increased proportional to the length of infection.	(160)	
*Citrobacter freundii*	Worm	polyQ	PolyQ^*b*^	Disrupted protein folding environment, which led to toxic polyQ aggregation.	(71)	
*Clostridium butyricum*	Mouse	APP/PS1	AD	Ameliorated cognitive defects and neurodegeneration, and attenuated Aβ deposition.	(161)	
*Enterococcus faecalis*	Worm	polyQ	PolyQ^*b*^	Enhanced polyQ aggregation.	(162)	
*Erwinia carotovora*	Fly	Aβ	AD	Increased vacuolar degeneration in the brain, impaired locomotion, and reduced lifespan.	(163)	
*Escherichia coli*	Fly	polyQ	PolyQ^*b*^	Increased polyQ aggregation, impaired mobility, and decreased lifespan.	(164)	
*Escherichia coli*	Worm	polyQ	PolyQ^*b*^	Disrupted protein folding environment, which led to toxic polyQ aggregation.	(71)	
*Escherichia coli*	Mouse	APP (Tg2576)	AD	Impaired cognition and led to necrosis of hippocampal neurons.	(165)	
*Klebsiella aerogenes*	Worm	polyQ	PolyQ^*b*^	Disrupted protein folding environment, which led to toxic polyQ aggregation.	(71)	
*Klebsiella oxytoca*	Worm	polyQ	PolyQ^*b*^	Disrupted protein folding environment, which led to toxic polyQ aggregation.	(71)	
*Klebsiella pneumoniae*	Worm	polyQ	PolyQ^*b*^	Disrupted protein folding environment, which led to toxic polyQ aggregation.	(71)	
*Lactobacillus casei*	Fly	Aβ	AD	Rescued the rough eye phenotype seen in flies with AD.	(166)	
*Lactobacillus fermentum*	Fly	Aβ	AD	Rescued the rough eye phenotype seen in flies with AD.	(166)	
*Lactobacillus fermentum^e^*	Fly	APP-BACE1	AD	Probiotic cocktail reduced the formation of Aβ plaques over time.	(77)	
*Lactococcus lactis^c^*	Mouse	MitoPark^*a*^	PD	Probiotic cocktail had neuroprotective effects on dopaminergic neurons and counteracted motor impairments.	(78)	
*Lactobacillus paracasei*	Fly	Aβ	AD	Suppressed eye degeneration.	(167)	
*Lactobacillus plantarum^e^*	Fly	APP-BACE1	AD	Reduced the formation of Aβ plaques.	(77)	
*Lactobacillus plantarum^c^*	Mouse	MitoPark^*a*^	PD	Neuroprotective effects on dopaminergic neurons and counteracted motor impairments.	(78)	
*Lactobacillus plantarum*	Rat	N/A	AD	Ameliorated cognitive defects and improved other pathological phenotypes otherwise induced by intraperitoneal injection of D-galactosidase, which induced AD phenotypes.	(79)	
*Lactobacillus plantarum*	Fly	Aβ	AD	Rescued the rough eye phenotype seen in flies with AD.	(166)	
*Lactobacillus reuteri^d^*	Rat	Aβ	AD	Improved memory and decreased Aβ plaques in rats that received hippocampal injections of Aβ.	(158)	
*Lactobacillus rhamnosis^c^*	Mouse	MitoPark^*a*^	PD	Probiotic cocktail had neuroprotective effects on dopaminergic neurons and counteracted motor impairments.	(78)	
*Lactobacillus rhamnosus^d^*	Rat	N/A	AD	Probiotic cocktail improved memory and decreased Aβ plaques in rats that received hippocampal injections of Aβ.	(158)	
*Lactobacillus rhamnosus^c^*	Mouse	MitoPark^*a*^	PD	Probiotic cocktail had neuroprotective effects on dopaminergic neurons and counteracted motor impairments.	(78)	
*Lactobacillus sakei*	Fly	Aβ	AD	Suppressed eye degeneration.	(167)	
*Porphyromonas gingivalis*	Mouse	APP	AD	Impaired cognitive function and increased Aβ deposition in the hippocampus and cortex.	(168)	
*Porphyromonas gingivalis*	Mouse	APP	AD	Increased Aβ deposition following intracerebral injection of live (but not heat-killed) bacteria.	(169)	
*Porphyromonas gingivalis*	Rat	N/A	AD	Encapsulated strains induced cognitive defects and increased Aβ levels as we as hippocampal tau hyperphosphorylation.	(170)	
*Prevotella corporis*	Worm	polyQ	PolyQ^*b*^	Attenuated polyQ aggregation and the associated toxicity.	(71)	
*Prevotella disiens*	Worm	polyQ	PolyQ^*b*^	Attenuated polyQ aggregation and the associated toxicity.	(71)	
*Proteus mirabilis*	Worm	polyQ	PolyQ^*b*^	Disrupted protein folding environment, which led to toxic polyQ aggregation.	(71)	
*Proteus mirabilis*	Mouse	N/A	PD	Impaired motility and α-synuclein aggregation in the brain and colon.	(149)	
*Pseudomonas aeruginosa*	Worm	polyQ	PolyQ^*b*^	Disrupted protein folding environment, which led to toxic polyQ aggregation.	(71, 72)	
*Pseudomonas aeruginosa*	Mouse	Amyloid/Tau	AD	Pneumonia initiates amyloid and tauopathy production by lung endothelium.	(171)	
*Pseudomonas entomophila*	Fly	Aβ	AD	Increased vacuolar degeneration in the brain, impaired locomotion, and reduced lifespan.	(163)	
*Salmonella enterica*	Worm	polyQ	PolyQ^*b*^	Disrupted protein folding environment, which led to toxic polyQ aggregation.	(71)	
*Salmonella* Typhimurium	Mouse	APP/PS1 (5XFAD)	AD	Enhanced and colocalized with Aβ deposits.	(172)	
*Shigella sonnei*	Worm	polyQ	PolyQ^*b*^	Disrupted protein folding environment, which led to toxic polyQ aggregation.	(71)	

^*a*^ Dopaminergic-specific absence of TFAM results in recapitulation of PD phenotypes. MitoPark is a genetic model for PD in which dopaminergic neurons selectively lack the transcription factor TFAM (mitochondrial transcription factor A), culminating in the symptoms and phenotypes present in PD.

^*b*^ PolyQ was used as a sensor of proteostasis but also models CAG repeat diseases such as HD, which are characterized by abnormally long stretches of glutamine "Q" repeats.

^*c*^ Bacterial strain was part of a probiotic cocktail that consisted of six bacterial strains: *Bifidobacterium bifidum*, *Bifidobacterium longum*, *Lactobacillus rhamnosis*, *Lactobacillus rhamnosus*, *Lactobacillus plantarum,* and *Lactococcus lactis*.

^*d*^ Bacterial strain was part of a probiotic cocktail that consisted of three bacterial strains: *Lactobacillus reuteri*, *Lactobacillus rhamnosus*, and *Bifidobacterium infantis*.

^*e*^ Bacterial strain was part of a probiotic cocktail that consisted of three bacterial strains: *Lactobacillus plantarum*, *Lactobacillus fermentum*, and *Bifidobacteria longum*.

^*f*^ Alzheimer’s disease (AD), Parkinson’s disease (PD), Huntington’s disease (HD), or amyotrophic lateral sclerosis (ALS). Aβ, amyloid-beta; APP, amyloid precursor protein; BACE-1, beta-site amyloid precursor protein cleaving enzyme 1; FAD, familial Alzheimer disease; N/A, not applicable; polyQ, polyglutamine; PS1, presenilin-1; TFAM, mitochondrial transcription factor A. "Worm" is used in place of *Caenorhabditis elegans*; "Fly," *Drosophila melanogaster*; "Mouse," *Mus musculus*; and "Rat," *Rattus norvegicus.*

Over 50% of infections among nursing home residents were due to MDR bacteria, indicating a high prevalence of antibiotic resistance present within the aged and predisposed population (173). It is estimated that nearly 70% of Americans with dementia will die in nursing homes (174). Recurrent infections and, consequently, antibiotic use among nursing home dementia patients are also frequent (175). In fact, long-term care residents are commonly colonized by MDR gram-negative bacteria, which are known to be proteotoxic, and such colonization is strongly associated with advanced dementia (176). Therefore, with nursing homes being hotspots for MDR bacteria, the overuse of antibiotics likely fuels the enrichment of the resistant gram-negative proteotoxic bacteria that further contribute to dementia and other neurological conditions. In support of this statement, a recent study used metagenomic sequencing to investigate the presence of AMR genes in the microbiota of a group of patients with advanced dementia and found a link between resistance gene density and the relative abundance of *P. mirabilis*, *Enterococcus faecalis*, and *Escherichia coli* (177). The authors further demonstrated that these strains had more resistance genes than many commensal bacteria in the gut. Perhaps not surprisingly, these bacteria are also associated with an increased risk of PCDs (Table 1). For example, a study found that people with PD have an increased presence of *Enterococcus* (178), and *E. faecalis* increased toxic polyQ aggregation in *C. elegans* (162) (Table 1). LPS from *E. coli* was found to be abundant in brains of AD patients (179), and *Escherichia-Shigella* abundance was found to be significantly increased in PD patients (178). Pathogenic strains of *E. coli* have also been shown to impair mobility in a polyQ *Drosophila* model and cognition in AD mice (164, 165) (Table 1). As previously mentioned, *P. mirabilis* was also associated with PD in humans (148).

*Porphyromonas gingivalis* is commonly associated with oral infections but can also colonize asymptomatically, as it was found in up to 25% of people with no oral disease (180). *P. gingivalis* products were found in the brains of AD patients, and the bacterium was shown to enhance the pathogenesis of the disease in mice (181) (Table 1). Injections of encapsulated strains of *P. gingivalis* in the palatal mucosa of rats led to memory defects, increased Aβ levels, and tau hypophosphorylation in the hippocampus, and intracerebral injection of *P. gingivalis* increased Aβ deposition in mice (169, 170). Similar findings were also present in another mouse study where oral inoculation of *P. gingivalis* impaired cognitive function and increased levels of Aβ deposition in the hippocampus and cortex of AD mice (168) (Table 1). Broad-spectrum antibiotics rarely eradicate this bacterium, which may lead to the emergence of and selection for MDR strains (182), increasing the concern that antibiotic use can enrich for its presence. The enrichment of these disease-associated bacteria becomes particularly concerning for people who might be asymptomatically colonized and are already at risk for PCDs.

**Fig 2:**
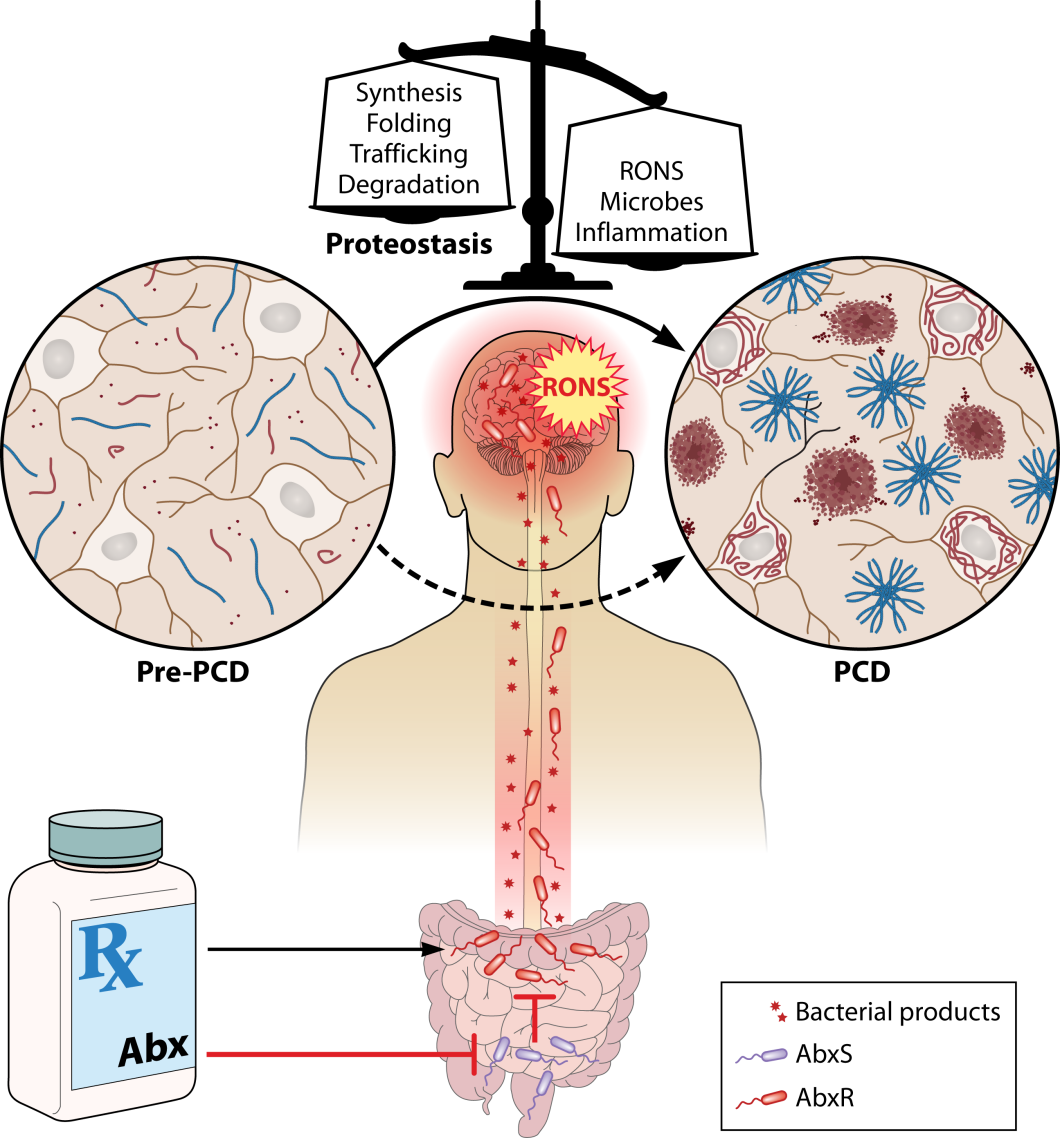
The effect of antibiotics on neurodegenerative protein conformational diseases. Antimicrobial agents kill commensal antibiotic-susceptible bacteria (AbS) that suppress proteotoxic antibiotic-resistant bacteria (AbR) that are thought to secrete products or signals that disrupt protein folding via the gut-brain axis. Bacteria can affect the pathogenesis of PCDs through direct or indirect interactions. Direct: Bacteria and their products can directly modulate host proteostasis (protein synthesis, folding, trafficking, or degradation). Indirect: Bacteria non-specifically induce inflammation and the generation of reactive oxygen and nitrogen species (RONS), which further disrupt host proteostasis.

Accumulating evidence from patient data suggests that *Chlamydia pneumoniae* might play a role in AD pathogenesis (183). Among perhaps the most convincing evidence is that *C. pneumoniae* DNA was found in ~90% of AD brains compared to ~5% of otherwise healthy controls (184). Intranasal inoculation of the bacterium led to Aβ deposition in the brains of infected mice (160) (Table 1). *C. pneumoniae* is a causative agent of pneumonia but likely exists asymptomatically in the majority of cases, as 50% of 20-year-olds have antibodies to the bacterium, which increases to 75% in 60–70-year-olds, and due to a 3–5-year limited antibody response, it is thought to commonly infect and reinfect people throughout their lifetime (185). Hence, antibiotic use could fuel resistance in those who are asymptomatically colonized with the pathogen and enrich its abundance (Fig. 2), as *in vitro* evidence supports the emergence of AMR after exposure to subinhibitory antibiotic concentrations (186).

## MODEL ORGANISMS FOR NEURODEGENERATIVE PCDS

Common organisms employed to model neurodegenerative PCDs include the nematode *Caenorhabditis elegans,* the fruit-fly *Drosophila melanogaster,* and rodents such as rats and mice. The fact that *C. elegans* can easily be colonized by a single bacterial species has made the model an attractive tool for studying host-bacteria interactions. Additionally, its neuronal network has been fully mapped, and its evolutionary conserved protein homeostasis networks have afforded the production of conclusive literature on the physiological processes that relate to general eukaryotic protein folding (187). Hence, *C. elegans* is arguably one of the most suitable model organisms to study the effect of bacteria on protein-folding diseases. Relative to *C. elegans*, *D. melanogaster* is a higher-order organism and has a brain and is thus capable of more sophisticated behavior, allowing for brain histopathological analyses as well as rudimentary assessments of cognitive and motor function. The higher complexity of *D. melanogaster* compared to *C. elegans* can also be a disadvantage; for example, *C. elegans* is transparent and allows for the detection of fluorescent reporters and sensors in real-time, and similar experiments in *C. elegans* and *D. melanogaster* are likely less labor-intensive in the former. Due to their invertebrate classification, neither flies nor nematodes have animal welfare restrictions. Rodent models are advantageous in that their physiology is closely related to humans; however, they are expensive, and experiments are lengthy and labor-intensive, particularly when studying age-associated diseases as they have a much longer lifespan. Furthermore, rodents do not seem to be a suitable model to identify PCD therapeutics given a 100% failure rate in humans (52). However, numerous studies, including those using rodents, have used animal models to link different bacteria to PCD pathogenicity. This section summarizes some of the results of the literature listed in Table 1, though not all of the information in Table 1 is elaborated here. Some of these bacteria and their connections to PCDs are discussed in more detail in previous sections.

One of the characteristics of all neurodegenerative PCDs is a deficiency in the cellular ability to maintain proteins that have a high propensity for aggregation in their folded, non-toxic state; as such, PCDs can be collectively referred to as disorders of proteostasis. Transgenic *C. elegans* that express fluorescent polyQ reporters exhibit quantifiable aggregates in response to proteotoxic stress (71, 162, 188, 189). Using these strains as sensors capable of detecting changes in polyQ aggregation has allowed for the discovery of microbes and microbial products that affect cellular proteostasis (71, 72, 190). *P. aeruginosa* was among the microbes that robustly disrupted host proteostasis and led to polyQ aggregation (71). In addition to *Pseudomonas* being linked to PCDs in humans and in a mouse model (145, 171, 191 – 194), *Pseudomonas entomophila* also aggravated disease-associated phenotypes in an AD fly model (163). Also consistent with the results obtained in worms demonstrating that *Salmonella enterica* Typhimurium disrupts host proteostasis, it was found that this microbe drove amyloid deposition in AD mice (172). The agreement in the microbial contribution to PCDs between humans and animal models and within models is also evident for other bacteria. For example, in addition to the aforementioned role of *P. mirabilis* in PD patients, the organism also contributed to the pathogenicity of PCDs in animal models, including worms and mice (71, 149). Pathogenic *E. coli* disrupted *C. elegans* proteostasis and caused disease-associated phenotypes in an HD fly and AD mouse models (71, 164, 165). In another study, a *C. elegans* polyQ model was used to show that *E. faecalis* disrupted host proteostasis as assessed by the enhancement of the polyQ aggregation (162). *E. faecalis* is normally part of the commensal microbiota, but in the event of gut dysbiosis, it can become enriched, compromise the intestinal barrier, and manifest in infection (195). As mentioned in the previous section, damage to the intestinal epithelium can enhance bacterial displacement, which could explain a significant increase in microbial presence in AD patients’ brains (24, 196).

Gut dysbiosis is implicated in a variety of diseases and is widely documented in people with PCDs (197). Numerous studies in humans and various animal models have used bacteria to induce or reverse gut dysbiosis to further examine the effect of microbial imbalance on neurodegenerative disease. For example, *Erwinia carotovora* was used to induce gut dysbiosis in AD flies to examine the effects of gut dysbiosis on AD phenotypes (163). The authors chose this bacterium due to the fact that it is non-pathogenic but can still persist in and colonize the gut. As such, they argued that the microbial dysbiosis caused by the bacterium aggravated AD phenotypes such that similar results were found using the pathogenic *P. entomophila*. Studies have also used bacteria to reverse gut dysbiosis, as consumption of probiotic bacteria is thought to help restore gut eubiosis, and *Bifidobacteria* and *Lactobacillus* are among common examples that are also known to affect cognitive function in humans (198, 199). Lee et al. have shown that *Bifidobacterium longum* can alleviate cognitive defects in AD mice, and this positive effect was associated with its restoration of microbial symbiosis in the gut (159). *B. longum* has been used in a probiotic cocktail in two separate studies that demonstrated positive effects on AD and PD pathology in flies and mice, respectively (77, 78). Additional evidence for the protective role of *Bifidobacterium* spp. comes from studies showing their suppressive effect against PD in flies and AD in rodents (77, 156 – 158, 200). Other beneficial bacteria come from the *Lactobacillus* genus and are associated with positive outcomes in a PD mouse model (78), AD fly models (166, 167), and an AD rat model (79).

While *L. plantarum* has the capacity to produce butyrate, a short-chain fatty acid that was shown to suppress protein aggregation, it is also known to enrich for butyrogenic bacteria in the swine (71, 201, 202). Butyrate was shown to restore gut eubiosis and convey neuroprotection in ALS mice (203). Walker et al. demonstrated that exogenous butyrate supplementation, as well as bacteria engineered to overproduce butyrate, can be beneficial to *C. elegans* proteostasis as assessed by reduced polyQ aggregation and the associated toxicity (71). In addition to *L. plantarum*, *Bacillus subtilis* was also shown to increase butyrate-producing bacteria (204). However, its role in protecting against neurotoxicity is likely more direct as its colonization of the *C. elegans* intestine increased lifespan, inhibited and reversed α-synuclein aggregation, and alleviated motility defects and other AD-associated phenotypes (154, 155, 205). Interestingly, *B. subtilis* is used to ferment soy products, such as Japanese *natto*, which is known for its neuroprotective properties (206, 207). *Akkermansia muciniphila* has emerged as the sentinel microbe that promotes intestinal permeability and growth of butyrogenic bacteria (208). While the bacterium is known for its numerous health benefits, including attenuation of cognitive defects and Aβ plaques in AD rodent models, its presence in PCD patients, predominantly PD, correlates with the pathogenicity (152, 153, 209, 210). Microbial communities rich in butyrate-producing bacteria, such as *Agathobaculum butyriciproducens*, have been associated with a diet that consists of healthy, plant-based foods (211). *A. butyriciproducens* has been shown to improve AD- and PD-associated phenotypes in mice (150, 151). In general, butyrogenic bacteria, including *Clostridium butyricum*, have been associated with neuroprotective effects in animal models and humans (161, 212, 213).

## POSSIBLE MECHANISMS BY WHICH BACTERIA CONTRIBUTE TO NEURODEGENERATIVE PCDS

The precise mechanisms by which bacteria impact neurodegenerative PCDs are not known; however, based on well-studied host-microbe interactions, the affected pathways likely involve inflammatory responses and all aspects of host proteostasis ranging from protein synthesis to folding, trafficking, and degradation. The cross talk between inflammation and proteostasis can further exaggerate proteotoxicity where inflammation is known to disrupt proteostasis which in turn triggers inflammation (214). Furthermore, bacteria promote the production and release of host reactive oxygen and nitrogen species (RONS), both of which also trigger inflammation and disrupt proteostasis (Fig. 2) (215, 216). While the abovementioned responses are mainly triggered as part of the innate immune reaction to bacteria and, therefore, contribute to the disruption of host proteostasis non-specifically, other responses are microbe specific. For example, *P. aeruginosa* inhibits USP10, a host deubiquitinating enzyme involved in the clearance of cystic fibrosis transmembrane receptor, thus, affecting protein concentration and trafficking (217). *P. aeruginosa* modulates other host processes, including translation and protein folding (218, 219). The influence of *P. aeruginosa* on broad host processes demonstrates multiple potential mechanisms by which bacteria can affect host proteostasis and ultimately the stability of proteins associated with PCDs. Other bacteria were also found to directly regulate stress responses in the host. For example, *Chlamydia* activates IRE1α, a major mediator of the endoplasmic reticulum unfolded protein response, which in turn activates a cascade of key inflammatory molecules, including two pathogen recognition receptors (PRRs), NOD1 and NOD2, and NF-κB that are known to affect PCDs (220 – 222). Various types of PRRs that respond to bacterial pathogens, including toll-like receptors, nucleotide-binding oligomerization domain-like receptors, and absent in melanoma-2-like receptors are also implemented in inflammation, likely contributing to the pathogenicity of PCDs (223).

## CONCLUDING REMARKS

In this review, we summarized current research on the microbial contribution to neurodegenerative PCDs and propose that bacteria indiscriminately affect destabilized proteins that are encoded within its host proteome via direct and indirect mechanisms. As such, bacteria or their products will affect host proteins that are more prone to misfolding and aggregation. While certain bacterial species may more robustly affect protein folding homeostasis leading to proteotoxicity, the disease they contribute to solely depends on the extent of the metastable proteome and the capacity of the proteostasis network to buffer any destabilizing mutations.

Antibiotic stewardship is increasingly becoming the top priority for all One Health sectors (healthcare, agriculture, and the environment) because of the antibiotics’ role in the emergence of AMR. However, due to limited data and only an emerging understanding of the microbial pathogenesis in PCDs, no emphasis is put on antibiotic stewardship in terms of prevention and management strategies for these debilitating diseases. Increasing evidence suggests that antibiotics are not inert in terms of their role in the pathogenesis of PCDs, and prescribers should be aware of that when recommending or implementing antimicrobial treatment strategies, not only in PCD patients but also in elders, and the remaining population from all ages.

With the increasing prevalence of AMR, more bacteria become resistant, which, upon antibiotic treatment, will lead to increased enrichment of proteotoxic gram-negative species while at the same time eradicating the protective microbiota (Fig. 2). Therefore, AMR will inevitably affect the prevalence of PCDs.

The vast amount of the described published literature concentrates mainly on AD and PD. As such, we have a much better understanding of the microbial contribution to these two diseases than other PCDs. Based on our analysis of the literature, we believe that the sporadic forms of AD and PD, and perhaps other PCDs, could be preventable and possibly manageable by targeting the gut microbiota.

## References

[1] Gould K . 2016. Antibiotics: from prehistory to the present day. J Antimicrob Chemother 71:572–575. doi:10.1093/jac/dkv484doi: 26851273

[2] Hippius H , Neundörfer G . 2003. The discovery of Alzheimer’s diseased. Dialogues Clin Neurosci 5:101–108. doi:10.31887/DCNS.2003.5.1/hhippiusdoi: 22034141 PMC3181715

[3] James Parkinson . 2002. Member of the royal college of surgeons, an essay on the shaking palsy. J Neuropsychiatry Clin Neurosci 14:223–236. doi:10.1176/jnp.14.2.223doi: 11983801

[4] Bhattacharyya KB . 2016. The story of George Huntington and his disease. Ann Indian Acad Neurol 19:25–28. doi:10.4103/0972-2327.175425doi: 27011624 PMC4782548

[5] Glicksman MA . 2011. The preclinical discovery of amyotrophic lateral sclerosis drugs. Expert Opin Drug Discov 6:1127–1138. doi:10.1517/17460441.2011.628654doi: 22646982 PMC3367799

[6] Brumback RA , Leech RW . 1994. Alzheimer’s disease: pathophysiology and the hope for therapy. J Okla State Med Assoc 87:103–111.doi: 8195921

[7] Braak H , Rüb U , Gai WP , Del Tredici K . 2003. Idiopathic Parkinson’s disease: possible routes by which vulnerable neuronal types may be subject to Neuroinvasion by an unknown pathogen. J Neural Transm (Vienna) 110:517–536. doi:10.1007/s00702-002-0808-2doi: 12721813

[8] Daey Ouwens IM , Lens CE , Fiolet ATL , Ott A , Koehler PJ , Verhoeven WMA . 2015. Clinical presentation of general paralysis of the insane in a Dutch psychiatric hospital, 1924-1954. Eur Neurol 74:54–59. doi:10.1159/000435835doi: 26183784

[9] Lafond RE , Lukehart SA . 2006. Biological basis for syphilis. Clin Microbiol Rev 19:29–49. doi:10.1128/CMR.19.1.29-49.2006doi: 16418521 PMC1360276

[10] Miklossy J . 2015. Historic evidence to support a causal relationship between spirochetal infections and Alzheimer’s disease. Front Aging Neurosci 7:46. doi:10.3389/fnagi.2015.00046doi: 25932012 PMC4399390

[11] Miklossy J . 2011. Alzheimer’s disease - a neurospirochetosis. Analysis of the evidence following Koch’s and Hill’s criteria. J Neuroinflammation 8:90. doi:10.1186/1742-2094-8-90doi: 21816039 PMC3171359

[12] Ghanem KG . 2010. REVIEW: neurosyphilis: a historical perspective and review. CNS Neurosci Ther 16:e157–68. doi:10.1111/j.1755-5949.2010.00183.xdoi: 20626434 PMC6493817

[13] Valent P , Groner B , Schumacher U , Superti-Furga G , Busslinger M , Kralovics R , Zielinski C , Penninger JM , Kerjaschki D , Stingl G , Smolen JS , Valenta R , Lassmann H , Kovar H , Jäger U , Kornek G , Müller M , Sörgel F . 2016. Paul Ehrlich (1854-1915) and his contributions to the foundation and birth of translational medicine. J Innate Immun 8:111–120. doi:10.1159/000443526doi: 26845587 PMC6738855

[14] McGeer PL , Harada N , Kimura H , McGeer EG , Schulzer M . 1992. Prevalence of dementia amongst elderly Japanese with leprosy: apparent effect of chronic drug therapy. Dement Geriatr Cogn Disord 3:146–149. doi:10.1159/000107010

[15] Nicolson GL , Nasralla MY , Haier J , Pomfret J . 2002. High frequency of systemic mycoplasmal infections in Gulf war veterans and civilians with amyotrophic lateral sclerosis (ALS). J Clin Neurosci 9:525–529. doi:10.1054/jocn.2001.1075doi: 12383408

[16] Kim JS , Choi IS , Lee MC . 1995. Reversible parkinsonism and dystonia following probable mycoplasma pneumoniae infection. Mov Disord 10:510–512. doi:10.1002/mds.870100419doi: 7565836

[17] Kountouras J , Tsolaki M , Gavalas E , Boziki M , Zavos C , Karatzoglou P , Chatzopoulos D , Venizelos I . 2006. Relationship between Helicobacter pylori infection and Alzheimer disease. Neurology 66:938–940. doi:10.1212/01.wnl.0000203644.68059.5fdoi: 16567719

[18] Kountouras J , Boziki M , Gavalas E , Zavos C , Grigoriadis N , Deretzi G , Tzilves D , Katsinelos P , Tsolaki M , Chatzopoulos D , Venizelos I . 2009. Eradication of Helicobacter pylori may be beneficial in the management of Alzheimer’s disease. J Neurol 256:758–767. doi:10.1007/s00415-009-5011-zdoi: 19240960

[19] Chang Y-P , Chiu G-F , Kuo F-C , Lai C-L , Yang Y-H , Hu H-M , Chang P-Y , Chen C-Y , Wu D-C , Yu F-J . 2013. Eradication of Helicobacter pylori is associated with the progression of dementia: a population-based study. Gastroenterol Res Pract 2013:175729. doi:10.1155/2013/175729doi: 24371435 PMC3859120

[20] Fukuda Y , Bamba H , Okui M , Tamura K , Tanida N , Satomi M , Shimoyama T , Nishigami T . 2001. Helicobacter Pylori infection increases mucosal permeability of the stomach and intestine. Digestion 63 Suppl 1:93–96. doi:10.1159/000051918doi: 11173917

[21] McGee DJ , Lu X-H , Disbrow EA . 2018. Stomaching the possibility of a pathogenic role for Helicobacter pylori in Parkinson’s disease. J Parkinsons Dis 8:367–374. doi:10.3233/JPD-181327doi: 29966206 PMC6130334

[22] Dobbs SM , Dobbs RJ , Weller C , Charlett A . 2000. Link between Helicobacter pylori infection and idiopathic parkinsonism. Med Hypotheses 55:93–98. doi:10.1054/mehy.2000.1110doi: 10904422

[23] Zhang R , Miller RG , Gascon R , Champion S , Katz J , Lancero M , Narvaez A , Honrada R , Ruvalcaba D , McGrath MS . 2009. Circulating endotoxin and systemic immune activation in sporadic amyotrophic lateral sclerosis (sALS). J Neuroimmunol 206:121–124. doi:10.1016/j.jneuroim.2008.09.017doi: 19013651 PMC2995297

[24] Emery DC , Shoemark DK , Batstone TE , Waterfall CM , Coghill JA , Cerajewska TL , Davies M , West NX , Allen SJ . 2017. 16S rRNA next generation sequencing analysis shows bacteria in Alzheimer’s post-mortem brain. Front Aging Neurosci 9:195. doi:10.3389/fnagi.2017.00195doi: 28676754 PMC5476743

[25] Ambrosini YM , Borcherding D , Kanthasamy A , Kim HJ , Willette AA , Jergens A , Allenspach K , Mochel JP . 2019. The gut-brain axis in neurodegenerative diseases and relevance of the canine model: a review. Front Aging Neurosci 11:130. doi:10.3389/fnagi.2019.00130doi: 31275138 PMC6591269

[26] Shabayek S , Spellerberg B . 2018. Group B streptococcal colonization, molecular characteristics, and epidemiology. Front Microbiol 9:437. doi:10.3389/fmicb.2018.00437doi: 29593684 PMC5861770

[27] Soriani M , Santi I , Taddei A , Rappuoli R , Grandi G , Telford JL . 2006. Group B streptococcus crosses human epithelial cells by a paracellular route. J Infect Dis 193:241–250. doi:10.1086/498982doi: 16362888

[28] McKee DH , Sussman JD . 2005. Case report: severe acute parkinsonism associated with streptococcal infection and antibasal ganglia antibodies. Mov Disord 20:1661–1663. doi:10.1002/mds.20641doi: 16078204

[29] Kutty SK , Zainulabid UA . 2021. Acute parkinsonism in young adult following streptococcal infection. Bangladesh J Infect Dis 7:110–112. doi:10.3329/bjid.v7i2.51522

[30] Ben-Pazi H , Livne A , Shapira Y , Dale RC . 2003. Parkinsonian features after streptococcal pharyngitis. J Pediatr 143:267–269. doi:10.1067/S0022-3476(03)00366-4doi: 12970645

[31] Kelly JR , Kennedy PJ , Cryan JF , Dinan TG , Clarke G , Hyland NP . 2015. Breaking down the barriers: the gut microbiome, intestinal permeability and stress-related psychiatric disorders. Front Cell Neurosci 9:392. doi:10.3389/fncel.2015.00392doi: 26528128 PMC4604320

[32] Batista CRA , Gomes GF , Candelario-Jalil E , Fiebich BL , de Oliveira ACP . 2019. Lipopolysaccharide-induced neuroinflammation as a bridge to understand neurodegeneration. Int J Mol Sci 20:2293. doi:10.3390/ijms20092293doi: 31075861 PMC6539529

[33] Brown GC . 2019. The endotoxin hypothesis of neurodegeneration. J Neuroinflammation 16:180. doi:10.1186/s12974-019-1564-7doi: 31519175 PMC6744684

[34] Glaros TG , Chang S , Gilliam EA , Maitra U , Deng H , Li L . 2013. Causes and consequences of low grade endotoxemia and inflammatory diseases. Front Biosci (Schol Ed) 5:754–765. doi:10.2741/s405doi: 23277084

[35] Zhan X , Stamova B , Sharp FR . 2018. Lipopolysaccharide associates with amyloid plaques, neurons and oligodendrocytes in Alzheimer’s disease brain: a review. Front Aging Neurosci 10:42. doi:10.3389/fnagi.2018.00042doi: 29520228 PMC5827158

[36] Gorecki AM , Preskey L , Bakeberg MC , Kenna JE , Gildenhuys C , MacDougall G , Dunlop SA , Mastaglia FL , Akkari PA , Koengten F , Anderton RS . 2019. Altered gut microbiome in Parkinson’s disease and the influence of lipopolysaccharide in a human Α-synuclein over-expressing mouse model. Front Neurosci 13:839. doi:10.3389/fnins.2019.00839doi: 31440136 PMC6693556

[37] Deng I , Corrigan F , Zhai G , Zhou X-F , Bobrovskaya L . 2020. Lipopolysaccharide animal models of Parkinson’s disease: recent progress and relevance to clinical disease. Brain Behav Immun Health 4:100060. doi:10.1016/j.bbih.2020.100060doi: 34589845 PMC8474547

[38] Gautieri A , Beeg M , Gobbi M , Rigoldi F , Colombo L , Salmona M . 2019. The anti-amyloidogenic action of doxycycline: a molecular dynamics study on the interaction with Aβ42. Int J Mol Sci 20:4641. doi:10.3390/ijms20184641doi: 31546787 PMC6769662

[39] De Luigi A , Mariani A , De Paola M , Re Depaolini A , Colombo L , Russo L , Rondelli V , Brocca P , Adler-Abramovich L , Gazit E , Del Favero E , Cantù L , Salmona M . 2015. Doxycycline hinders phenylalanine fibril assemblies revealing a potential novel therapeutic approach in phenylketonuria. Sci Rep 5:15902. doi:10.1038/srep15902doi: 26510963 PMC4625134

[40] Cardoso I , Merlini G , Saraiva MJ . 2003. 4'-Iodo-4'-deoxydoxorubicin and tetracyclines disrupt transthyretin amyloid fibrils in vitro producing noncytotoxic species: screening for TTR fibril disrupters. FASEB J 17:803–809. doi:10.1096/fj.02-0764comdoi: 12724338

[41] Forloni G , Colombo L , Girola L , Tagliavini F , Salmona M . 2001. Anti-amyloidogenic activity of tetracyclines: studies in vitro. FEBS Lett 487:404–407. doi:10.1016/s0014-5793(00)02380-2doi: 11163366

[42] Loeb MB , Molloy DW , Smieja M , Standish T , Goldsmith CH , Mahony J , Smith S , Borrie M , Decoteau E , Davidson W , McDougall A , Gnarpe J , O’DONNell M , Chernesky M . 2004. A randomized, controlled trial of doxycycline and rifampin for patients with Alzheimer’s disease. J Am Geriatr Soc 52:381–387. doi:10.1111/j.1532-5415.2004.52109.xdoi: 14962152

[43] Mertsalmi TH , Pekkonen E , Scheperjans F . 2020. Antibiotic exposure and risk of Parkinson’s disease in Finland: a nationwide case-control study. Mov Disord 35:431–442. doi:10.1002/mds.27924doi: 31737957

[44] Sun J , Zhan Y , Mariosa D , Larsson H , Almqvist C , Ingre C , Zagai U , Pawitan Y , Fang F . 2019. Antibiotics use and risk of amyotrophic lateral sclerosis in Sweden. Eur J Neurol 26:1355–1361. doi:10.1111/ene.13986doi: 31087715

[45] Tuk B , Jousma H , Gaillard PJ . 2017. Treatment with penicillin G and hydrocortisone reduces ALS-associated symptoms: a case series of three patients. F1000Res 6:410. doi:10.12688/f1000research.10534.1doi: 28443187 PMC5383939

[46] Adams-Carr KL , Bestwick JP , Shribman S , Lees A , Schrag A , Noyce AJ . 2016. Constipation preceding Parkinson’s disease: a systematic review and meta-analysis. J Neurol Neurosurg Psychiatry 87:710–716. doi:10.1136/jnnp-2015-311680doi: 26345189

[47] Gordon PH , Moore DH , Miller RG , Florence JM , Verheijde JL , Doorish C , Hilton JF , Spitalny GM , MacArthur RB , Mitsumoto H , Neville HE , Boylan K , Mozaffar T , Belsh JM , Ravits J , Bedlack RS , Graves MC , McCluskey LF , Barohn RJ , Tandan R , Western ALS Study Group . 2007. Efficacy of minocycline in patients with amyotrophic lateral sclerosis: a phase III randomised trial. Lancet Neurol 6:1045–1053. doi:10.1016/S1474-4422(07)70270-3doi: 17980667

[48] Chopra I , Roberts M . 2001. Tetracycline antibiotics: mode of action, applications, molecular biology, and epidemiology of bacterial resistance. Microbiol Mol Biol Rev 65:232–260. doi:10.1128/MMBR.65.2.232-260.2001doi: 11381101 PMC99026

[49] Mortison JD , Schenone M , Myers JA , Zhang Z , Chen L , Ciarlo C , Comer E , Natchiar SK , Carr SA , Klaholz BP , Myers AG . 2018. Tetracyclines modify translation by targeting key human rRNA substructures. Cell Chem Biol 25:1506–1518. doi:10.1016/j.chembiol.2018.09.010doi: 30318461 PMC6309532

[50] Cankaya S , Cankaya B , Kilic U , Kilic E , Yulug B . 2019. The therapeutic role of minocycline in Parkinson’s disease. Drugs Context 8:212553. doi:10.7573/dic.212553doi: 30873213 PMC6408180

[51] Garrido-Mesa N , Zarzuelo A , Gálvez J . 2013. Minocycline: far beyond an antibiotic. Br J Pharmacol 169:337–352. doi:10.1111/bph.12139doi: 23441623 PMC3651660

[52] Mullane K , Williams M . 2019. Preclinical models of Alzheimer’s disease: relevance and translational validity. Curr Protoc Pharmacol 84:e57. doi:10.1002/cpph.57doi: 30802363

[53] Kieser S , Zdobnov EM , Trajkovski M . 2022. Comprehensive mouse microbiota genome catalog reveals major difference to its human counterpart. PLoS Comput Biol 18:e1009947. doi:10.1371/journal.pcbi.1009947doi: 35259160 PMC8932566

[54] Mehta RS , Lochhead P , Wang Y , Ma W , Nguyen LH , Kochar B , Huttenhower C , Grodstein F , Chan AT . 2022. Association of midlife antibiotic use with subsequent cognitive function in women. PLoS One 17:e0264649. doi:10.1371/journal.pone.0264649doi: 35320274 PMC8942267

[55] Sun J , Ludvigsson JF , Ingre C , Piehl F , Wirdefeldt K , Zagai U , Ye W , Fang F . 2022. Hospital-treated infections in early- and mid-life and risk of Alzheimer’s disease, Parkinson’s disease, and amyotrophic lateral sclerosis: a nationwide nested case-control study in Sweden. PLoS Med 19:e1004092. doi:10.1371/journal.pmed.1004092doi: 36107840 PMC9477309

[56] Kim M , Park SJ , Choi S , Chang J , Kim SM , Jeong S , Park YJ , Lee G , Son JS , Ahn JC , Park SM . 2022. Association between antibiotics and dementia risk: a retrospective cohort study. Front Pharmacol 13:888333. doi:10.3389/fphar.2022.888333doi: 36225572 PMC9548656

[57] Hertzberg VS , Singh H , Fournier CN , Moustafa A , Polak M , Kuelbs CA , Torralba MG , Tansey MG , Nelson KE , Glass JD . 2022. Gut microbiome differences between amyotrophic lateral sclerosis patients and spouse controls. Amyotroph Lateral Scler Frontotemporal Degener 23:91–99. doi:10.1080/21678421.2021.1904994doi: 33818222 PMC10676149

[58] Gerhardt S , Mohajeri MH . 2018. Changes of colonic bacterial composition in Parkinson’s disease and other neurodegenerative diseases. Nutrients 10:708. doi:10.3390/nu10060708doi: 29857583 PMC6024871

[59] Scheperjans F , Aho V , Pereira PAB , Koskinen K , Paulin L , Pekkonen E , Haapaniemi E , Kaakkola S , Eerola-Rautio J , Pohja M , Kinnunen E , Murros K , Auvinen P . 2015. Gut microbiota are related to Parkinson’s disease and clinical phenotype. Mov Disord 30:350–358. doi:10.1002/mds.26069doi: 25476529

[60] Jin M , Li J , Liu F , Lyu N , Wang K , Wang L , Liang S , Tao H , Zhu B , Alkasir R . 2019. Analysis of the gut microflora in patients with Parkinson’s disease. Front Neurosci 13:1184. doi:10.3389/fnins.2019.01184doi: 31824239 PMC6883725

[61] Hopfner F , Künstner A , Müller SH , Künzel S , Zeuner KE , Margraf NG , Deuschl G , Baines JF , Kuhlenbäumer G . 2017. Gut microbiota in Parkinson disease in a northern German cohort. Brain Res 1667:41–45. doi:10.1016/j.brainres.2017.04.019doi: 28506555

[62] Barichella M , Severgnini M , Cilia R , Cassani E , Bolliri C , Caronni S , Ferri V , Cancello R , Ceccarani C , Faierman S , Pinelli G , De Bellis G , Zecca L , Cereda E , Consolandi C , Pezzoli G . 2019. Unraveling gut microbiota in Parkinson’s disease and atypical Parkinsonism. Mov Disord 34:396–405. doi:10.1002/mds.27581doi: 30576008

[63] Aho VTE , Pereira PAB , Voutilainen S , Paulin L , Pekkonen E , Auvinen P , Scheperjans F . 2019. Gut microbiota in Parkinson’s disease: temporal stability and relations to disease progression. EBioMedicine 44:691–707. doi:10.1016/j.ebiom.2019.05.064doi: 31221587 PMC6606744

[64] Petrov VA , Saltykova IV , Zhukova IA , Alifirova VM , Zhukova NG , Dorofeeva YB , Tyakht AV , Kovarsky BA , Alekseev DG , Kostryukova ES , Mironova YS , Izhboldina OP , Nikitina MA , Perevozchikova TV , Fait EA , Babenko VV , Vakhitova MT , Govorun VM , Sazonov AE . 2017. Analysis of gut microbiota in patients with Parkinson’s disease. Bull Exp Biol Med 162:734–737. doi:10.1007/s10517-017-3700-7doi: 28429209

[65] Bedarf JR , Hildebrand F , Coelho LP , Sunagawa S , Bahram M , Goeser F , Bork P , Wüllner U . 2017. Functional implications of microbial and viral gut metagenome changes in early stage L-DOPA-Naïve Parkinson’s disease patients. Genome Med 9:61. doi:10.1186/s13073-017-0451-zdoi: 28662719 PMC5492116

[66] Mertsalmi TH , Aho VTE , Pereira PAB , Paulin L , Pekkonen E , Auvinen P , Scheperjans F . 2017. More than constipation - bowel symptoms in Parkinson’s disease and their connection to gut microbiota. Eur J Neurol 24:1375–1383. doi:10.1111/ene.13398doi: 28891262

[67] Shen L , Liu L , Ji H-F . 2017. Alzheimer’s disease histological and nbsp;behavioral manifestations in and nbsp;transgenic mice correlate with and nbsp;specific and nbsp;gut microbiome state. J Alzheimers Dis 56:385–390. doi:10.3233/JAD-160884doi: 27911317

[68] Savica R , Carlin JM , Grossardt BR , Bower JH , Ahlskog JE , Maraganore DM , Bharucha AE , Rocca WA . 2009. Medical records documentation of constipation preceding Parkinson disease: a case-control study. Neurology 73:1752–1758. doi:10.1212/WNL.0b013e3181c34af5doi: 19933976 PMC2788809

[69] Secretariat G . 2022. GBIF secretariat: GBIF backbone taxonomy. Available from: https://www.gbif.org/species/5284517

[70] Garrett WS . 2015. Edited by J. E. Bennett , R. Dolin , and M. J. Blaser . 249 - Bacteroides, prevotella, porphyromonas, and Fusobacterium species (and other medically important anaerobic gram-negative bacilli), in mandell, douglas, and bennett’s principles and practice of infectious diseases. 8th ed, p 2773–2780. W.B. Saunders, Philadelphia.

[71] Walker AC , Bhargava R , Vaziriyan-Sani AS , Pourciau C , Donahue ET , Dove AS , Gebhardt MJ , Ellward GL , Romeo T , Czyż DM . 2021. Colonization of the caenorhabditis elegans gut with human enteric bacterial pathogens leads to proteostasis disruption that is rescued by butyrate. PLoS Pathog 17:e1009510. doi:10.1371/journal.ppat.1009510doi: 33956916 PMC8101752

[72] Walker AC , Bhargava R , Dove AS , Brust AS , Owji AA , Czyż DM . 2022. Bacteria-derived protein aggregates contribute to the disruption of host proteostasis. Int J Mol Sci 23:4807. doi:10.3390/ijms23094807doi: 35563197 PMC9103901

[73] Guo L , Zhang D , Fu S , Zhang J , Zhang X , He J , Peng C , Zhang Y , Qiu Y , Ye C , Liu Y , Wu Z , Hu C-AA . 2021. Metagenomic sequencing analysis of the effects of colistin sulfate on the pig gut microbiome. Front Vet Sci 8:663820. doi:10.3389/fvets.2021.663820doi: 34277753 PMC8282896

[74] Liu C , Cheng X , Zhong S , Liu Z , Liu F , Lin X , Zhao Y , Guan M , Xiao T , Jolkkonen J , Wang Y , Zhao C . 2022. Long-term modification of gut microbiota by broad-spectrum antibiotics improves stroke outcome in rats. Stroke Vasc Neurol 7:381–389. doi:10.1136/svn-2021-001231doi: 35577395 PMC9614136

[75] Wang T , Hu X , Liang S , Li W , Wu X , Wang L , Jin F . 2015. Lactobacillus fermentum NS9 restores the antibiotic induced physiological and psychological abnormalities in rats. Benef Microbes 6:707–717. doi:10.3920/BM2014.0177doi: 25869281

[76] Westfall S , Lomis N , Kahouli I , Dia SY , Singh SP , Prakash S . 2017. Microbiome, probiotics and neurodegenerative diseases: deciphering the gut brain axis. Cell Mol Life Sci 74:3769–3787. doi:10.1007/s00018-017-2550-9doi: 28643167 PMC11107790

[77] Westfall S , Lomis N , Prakash S . 2019. A novel synbiotic delays Alzheimer’s disease onset via combinatorial gut-brain-axis signaling in Drosophila melanogaster. PLoS One 14:e0214985. doi:10.1371/journal.pone.0214985doi: 31009489 PMC6476497

[78] Hsieh T-H , Kuo C-W , Hsieh K-H , Shieh M-J , Peng C-W , Chen Y-C , Chang Y-L , Huang Y-Z , Chen C-C , Chang P-K , Chen K-Y , Chen H-Y . 2020. Probiotics alleviate the progressive deterioration of motor functions in a mouse model of Parkinson’s disease. Brain Sci 10:206. doi:10.3390/brainsci10040206doi: 32244769 PMC7226147

[79] Nimgampalle M , Kuna Y . 2017. Anti-alzheimer properties of probiotic, lactobacillus plantarum MTCC 1325 in Alzheimer’s disease induced Albino rats. J Clin Diagn Res 11:KC01–KC05. doi:10.7860/JCDR/2017/26106.10428doi: PMC5620801 28969160

[80] Akbari E , Asemi Z , Daneshvar Kakhaki R , Bahmani F , Kouchaki E , Tamtaji OR , Hamidi GA , Salami M . 2016. Effect of probiotic supplementation on cognitive function and metabolic status in Alzheimer’s disease: a randomized, double-blind and controlled Trial. Front Aging Neurosci 8:256. doi:10.3389/fnagi.2016.00256doi: 27891089 PMC5105117

[81] Wohlgemuth I , Garofalo R , Samatova E , Günenç AN , Lenz C , Urlaub H , Rodnina MV . 2021. Translation error clusters induced by aminoglycoside antibiotics. Nat Commun 12:1830. doi:10.1038/s41467-021-21942-6doi: 33758186 PMC7987974

[82] Bollen C , Dewachter L , Michiels J . 2021. Protein aggregation as a bacterial strategy to survive antibiotic treatment. Front Mol Biosci 8:669664. doi:10.3389/fmolb.2021.669664doi: 33937340 PMC8085434

[83] Sender R , Fuchs S , Milo R . 2016. Revised estimates for the number of human and bacteria cells in the body. PLOS Biol 14:e1002533. doi:10.1371/journal.pbio.1002533doi: 27541692 PMC4991899

[84] Gilbert JA , Blaser MJ , Caporaso JG , Jansson JK , Lynch SV , Knight R . 2018. Current understanding of the human microbiome. Nat Med 24:392–400. doi:10.1038/nm.4517doi: 29634682 PMC7043356

[85] Barnhart MM , Chapman MR . 2006. Curli biogenesis and function. Annu Rev Microbiol 60:131–147. doi:10.1146/annurev.micro.60.080805.142106doi: 16704339 PMC2838481

[86] Van Gerven N , Van der Verren SE , Reiter DM , Remaut H . 2018. The role of functional amyloids in bacterial virulence. J Mol Biol 430:3657–3684. doi:10.1016/j.jmb.2018.07.010doi: 30009771 PMC6173799

[87] Hall CW , Mah T-F . 2017. Molecular mechanisms of biofilm-based antibiotic resistance and tolerance in pathogenic bacteria. FEMS Microbiol Rev 41:276–301. doi:10.1093/femsre/fux010doi: 28369412

[88] Costerton JW , Stewart PS , Greenberg EP . 1999. Bacterial biofilms: a common cause of persistent infections. Science 284:1318–1322. doi:10.1126/science.284.5418.1318doi: 10334980

[89] Chen SG , Stribinskis V , Rane MJ , Demuth DR , Gozal E , Roberts AM , Jagadapillai R , Liu R , Choe K , Shivakumar B , Son F , Jin S , Kerber R , Adame A , Masliah E , Friedland RP . 2016. Exposure to the functional bacterial amyloid protein curli enhances alpha-synuclein aggregation in aged fischer 344 rats and Caenorhabditis elegans. Sci Rep 6:34477. doi:10.1038/srep34477doi: 27708338 PMC5052651

[90] Wang C , Lau CY , Ma F , Zheng C . 2021. Genome-wide screen identifies curli amyloid fibril as a bacterial component promoting host neurodegeneration. Proc Natl Acad Sci U S A 118:e2106504118. doi:10.1073/pnas.2106504118doi: 34413194 PMC8403922

[91] Sampson TR , Challis C , Jain N , Moiseyenko A , Ladinsky MS , Shastri GG , Thron T , Needham BD , Horvath I , Debelius JW , Janssen S , Knight R , Wittung-Stafshede P , Gradinaru V , Chapman M , Mazmanian SK . 2020. A gut bacterial amyloid promotes α-synuclein aggregation and motor impairment in mice. Elife 9:e53111. doi:10.7554/eLife.53111doi: 32043464 PMC7012599

[92] Brouillette J , Caillierez R , Zommer N , Alves-Pires C , Benilova I , Blum D , De Strooper B , Buée L . 2012. Neurotoxicity and memory deficits induced by soluble low-molecular-weight amyloid-Β1-42 oligomers are revealed in vivo by using a novel animal model. J Neurosci 32:7852–7861. doi:10.1523/JNEUROSCI.5901-11.2012doi: 22674261 PMC6620963

[93] Zhao J , Wen D . 2017. Pore-scale simulation of wettability and interfacial tension effects on flooding process for enhanced oil recovery. RSC Adv 7:41391–41398. doi:10.1039/c7ra07325adoi: 29308190 PMC5735360

[94] Christensen LFB , Jensen KF , Nielsen J , Vad BS , Christiansen G , Otzen DE . 2019. Reducing the amyloidogenicity of functional amyloid protein FapC increases its ability to inhibit α-synuclein fibrillation. ACS Omega 4:4029–4039. doi:10.1021/acsomega.8b03590doi: 31459612 PMC6647998

[95] Javed I , Zhang Z , Adamcik J , Andrikopoulos N , Li Y , Otzen DE , Lin S , Mezzenga R , Davis TP , Ding F , Ke PC . 2020. Accelerated amyloid beta pathogenesis by bacterial amyloid FapC. Adv Sci 7:2001299. doi:10.1002/advs.202001299doi: PMC7509637 32999841

[96] Saiman L , Siegel J , Cystic Fibrosis Foundation Consensus Conference on Infection Control Participants . 2003. Infection control recommendations for patients with cystic fibrosis: microbiology, important pathogens, and infection control practices to prevent patient-to-patient transmission. Am J Infect Control 31:S1–62.doi: 12762292

[97] Roy B , Woo MS , Vacas S , Eshaghian P , Rao AP , Kumar R . 2021. Regional brain tissue changes in patients with cystic fibrosis. J Transl Med 19:419. doi:10.1186/s12967-021-03092-xdoi: 34627274 PMC8502335

[98] Elce V , Del Pizzo A , Nigro E , Frisso G , Martiniello L , Daniele A , Elce A . 2020. Impact of physical activity on cognitive functions: a new field for research and management of cystic fibrosis. Diagnostics 10:489. doi:10.3390/diagnostics10070489doi: 32708398 PMC7400241

[99] Van Den Eeden SK , Tanner CM , Bernstein AL , Fross RD , Leimpeter A , Bloch DA , Nelson LM . 2003. Incidence of Parkinson’s disease: variation by age, gender, and race/ethnicity. Am J Epidemiol 157:1015–1022. doi:10.1093/aje/kwg068doi: 12777365

[100] Guerreiro R , Bras J . 2015. The age factor in Alzheimer’s disease. Genome Med 7:106. doi:10.1186/s13073-015-0232-5doi: 26482651 PMC4617238

[101] Simpson T , Elston C , Macedo P , Perrin F . 2019. Amyloidosis in cystic fibrosis. Paediatr Respir Rev 31:32–34. doi:10.1016/j.prrv.2019.04.007doi: 31288987

[102] Marhaug G , Permin H , Husby G . 1983. Amyloid-related serum protein (SAA) as an indicator of lung infection in cystic fibrosis. Acta Paediatr Scand 72:861–866. doi:10.1111/j.1651-2227.1983.tb09831.xdoi: 6673488

[103] Loikas D , Wettermark B , von Euler M , Bergman U , Schenck-Gustafsson K . 2013. Differences in drug utilisation between men and women: a cross-sectional analysis of all dispensed drugs in Sweden. BMJ Open 3:e002378. doi:10.1136/bmjopen-2012-002378doi: PMC3646185 23645921

[104] Smith DRM , Dolk FCK , Smieszek T , Robotham JV , Pouwels KB . 2018. Understanding the gender gap in antibiotic prescribing: a cross-sectional analysis of english primary care. BMJ Open 8:e020203. doi:10.1136/bmjopen-2017-020203doi: PMC5855331 29472269

[105] Alzheimer’s disease facts and figures. 2021. Alzheimers Dement 17:327–406. doi:10.1002/alz.12328doi: 33756057

[106] Chêne G , Beiser A , Au R , Preis SR , Wolf PA , Dufouil C , Seshadri S . 2015. Gender and incidence of dementia in the framingham heart study from mid-adult life. Alzheimers Dement 11:310–320. doi:10.1016/j.jalz.2013.10.005doi: 24418058 PMC4092061

[107] Ding T , Schloss PD . 2014. Dynamics and associations of microbial community types across the human body. Nature 509:357–360. doi:10.1038/nature13178doi: 24739969 PMC4139711

[108] Takagi T , Naito Y , Inoue R , Kashiwagi S , Uchiyama K , Mizushima K , Tsuchiya S , Dohi O , Yoshida N , Kamada K , Ishikawa T , Handa O , Konishi H , Okuda K , Tsujimoto Y , Ohnogi H , Itoh Y . 2019. Differences in gut microbiota associated with age, sex, and stool consistency in healthy Japanese subjects. J Gastroenterol 54:53–63. doi:10.1007/s00535-018-1488-5doi: 29926167

[109] Mueller S , Saunier K , Hanisch C , Norin E , Alm L , Midtvedt T , Cresci A , Silvi S , Orpianesi C , Verdenelli MC , Clavel T , Koebnick C , Zunft H-JF , Doré J , Blaut M . 2006. Differences in fecal microbiota in different European study populations in relation to age, gender, and country: a cross-sectional study. Appl Environ Microbiol 72:1027–1033. doi:10.1128/AEM.72.2.1027-1033.2006doi: 16461645 PMC1392899

[110] Europe A . 2021. Prevalence of dementia in Europe. Available from: https://www.alzheimer-europe.org/dementia/prevalence-dementia-europe

[111] Control, E.C.f.D.P.a . 2014. D.P.A., trends of consumption of antibacterials for systemic use (ATC group J01) in the community, EU/EEA countries 2008-2012

[112] European Medicines Agency, E.S.o.V.A.C ,. 2017. Sales of veterinary antimicrobial agents in 30 European countries in 2015. trends from 2010 to 2015

[113] Curelaru A , Marzolf SJ , Provost J-CKG , Zeon HHH . 2021. Social isolation in dementia: the effects of COVID-19. J Nurse Pract 17:950–953. doi:10.1016/j.nurpra.2021.05.002doi: 34658679 PMC8504102

[114] Kazawa K , Kubo T , Akishita M , Ishii S . 2022. Long-term impact of the COVID-19 pandemic on facility- and home-dwelling people with dementia: perspectives from professionals involved in dementia care. Geriatr Gerontol Int 22:832–838. doi:10.1111/ggi.14465doi: 36068077 PMC9538434

[115] Smaling HJA , Tilburgs B , Achterberg WP , Visser M . 2022. The impact of social distancing due to the COVID-19 pandemic on people with dementia,family carers and healthcare professionals:a qualitative study. Int J Environ Res Public Health 19:519. doi:10.3390/ijerph19010519doi: 35010779 PMC8744737

[116] Pierce J , Stevens MP . 2021. COVID-19 and antimicrobial stewardship: lessons learned, best practices, and future implications. Int J Infect Dis 113:103–108. doi:10.1016/j.ijid.2021.10.001doi: 34624517 PMC8491953

[117] Wang L , Davis PB , Volkow ND , Berger NA , Kaelber DC , Xu R . 2022. Association of COVID-19 with new-onset Alzheimer’s disease. J Alzheimers Dis 89:411–414. doi:10.3233/JAD-220717doi: 35912749 PMC10361652

[118] Alzheimer's Association . 2022. 2022 Alzheimer’s disease facts and figures. 10.1002/alz.12638 35289055

[119] Qureshi AI , Baskett WI , Huang W , Naqvi SH , Shyu C-R . 2022. New-onset dementia among survivors of pneumonia associated with severe acute respiratory syndrome coronavirus 2 infection. Open Forum Infect Dis 9:ofac115. doi:10.1093/ofid/ofac115doi: 35350170 PMC8903511

[120] Lin MT , Balczon R , Pittet J-F , Wagener BM , Moser SA , Morrow KA , Voth S , Francis CM , Leavesley S , Bell J , Alvarez DF , Stevens T . 2018. Nosocomial pneumonia elicits an endothelial proteinopathy: evidence for a source of neurotoxic amyloids in critically ill patients. Am J Respir Crit Care Med 198:1575–1578. doi:10.1164/rccm.201801-0060LEdoi: 30280916 PMC6298632

[121] Liu Y-H , Chen Y , Wang Q-H , Wang L-R , Jiang L , Yang Y , Chen X , Li Y , Cen Y , Xu C , Zhu J , Li W , Wang Y-R , Zhang L-L , Liu J , Xu Z-Q , Wang Y-J . 2022. One-year trajectory of cognitive changes in older survivors of COVID-19 in Wuhan, China: a longitudinal cohort study. JAMA Neurol 79:509–517. doi:10.1001/jamaneurol.2022.0461doi: 35258587 PMC8905512

[122] Rhodes CH , Priemer DS , Karlovich E , Perl DP , Goldman J . 2022. Β-Amyloid deposits in young COVID patients. SSRN Journal. doi:10.2139/ssrn.4003213 36264253

[123] Langford BJ , So M , Raybardhan S , Leung V , Soucy J-PR , Westwood D , Daneman N , MacFadden DR . 2021. Antibiotic prescribing in patients with COVID-19: rapid review and meta-analysis. Clin Microbiol Infect 27:520–531. doi:10.1016/j.cmi.2020.12.018doi: 33418017 PMC7785281

[124] Zarifkar P , Peinkhofer C , Benros ME , Kondziella D . 2022. Frequency of neurological diseases after COVID-19, influenza A/B and bacterial pneumonia. Front Neurol 13. doi:10.3389/fneur.2022.904796doi: PMC9259944 35812108

[125] Piekut T , Hurła M , Banaszek N , Szejn P , Dorszewska J , Kozubski W , Prendecki M . 2022. Infectious agents and Alzheimer’s disease. J Integr Neurosci 21:73. doi:10.31083/j.jin2102073doi: 35364661

[126] Niklasson B , Lindquist L , Klitz W , Englund E , Netherlands Brain Bank . 2020. Picornavirus identified in Alzheimer’s disease brains: a pathogenic path? J Alzheimers Dis Rep 4:141–146. doi:10.3233/ADR-200174doi: 32587947 PMC7306919

[127] Niklasson B , Lindquist L , Klitz W , Fredrikson S , Morgell R , Mohammadi R Netherlands Brain Bank Karapetyan Y , Englund E . 2022. Picornavirus may be linked to Parkinson&rsquo;s disease through viral antigen in dopamine-containing neurons of substantia nigra. Microorganisms 10:599. doi:10.3390/microorganisms10030599doi: 35336174 PMC8953350

[128] Aviner R , Frydman J . 2020. Proteostasis in viral infection: Unfoulding the complex virus-chaperone interplay. Cold Spring Harb Perspect Biol 12:a034090. doi:10.1101/cshperspect.a034090doi: 30858229 PMC7050591

[129] Lubkowska A , Pluta W , Strońska A , Lalko A . 2021. Role of heat shock proteins (HSP70 and HSP90) in viral Infection. Int J Mol Sci 22:9366. doi:10.3390/ijms22179366doi: 34502274 PMC8430838

[130] Doll JR , Hoebe K , Thompson RL , Sawtell NM . 2020. Resolution of herpes simplex virus reactivation in vivo results in neuronal destruction. PLoS Pathog 16:e1008296. doi:10.1371/journal.ppat.1008296doi: 32134994 PMC7058292

[131] Zhou L , Miranda-Saksena M , Saksena NK . 2013. Viruses and neurodegeneration. Virol J 10:172. doi:10.1186/1743-422X-10-172doi: 23724961 PMC3679988

[132] Lange K , Buerger M , Stallmach A , Bruns T . 2016. Effects of antibiotics on gut microbiota. Dig Dis 34:260–268. doi:10.1159/000443360doi: 27028893

[133] Engen PA , Green SJ , Voigt RM , Forsyth CB , Keshavarzian A . 2015. The gastrointestinal microbiome: alcohol effects on the composition of intestinal microbiota. Alcohol Res 37:223–236.doi: 26695747 10.35946/arcr.v37.2.07 PMC4590619

[134] Marques C , Dinis L , Barreiros Mota I , Morais J , Ismael S , Pereira-Leal JB , Cardoso J , Ribeiro P , Beato H , Resende M , Espírito Santo C , Cortez AP , Rosário A , Pestana D , Teixeira D , Faria A , Calhau C . 2022. Impact of beer and nonalcoholic beer consumption on the gut microbiota: a randomized, double-blind, controlled trial. J Agric Food Chem 70:13062–13070. doi:10.1021/acs.jafc.2c00587doi: 35834180 PMC9776556

[135] Huang C , Shi G . 2019. Smoking and microbiome in oral, airway, gut and some systemic diseases. J Transl Med 17:225. doi:10.1186/s12967-019-1971-7doi: 31307469 PMC6632217

[136] Monda V , Villano I , Messina A , Valenzano A , Esposito T , Moscatelli F , Viggiano A , Cibelli G , Chieffi S , Monda M , Messina G . 2017. Exercise modifies the gut microbiota with positive health effects. Oxid Med Cell Longev 2017:3831972. doi:10.1155/2017/3831972doi: 28357027 PMC5357536

[137] Singh RK , Chang H-W , Yan D , Lee KM , Ucmak D , Wong K , Abrouk M , Farahnik B , Nakamura M , Zhu TH , Bhutani T , Liao W . 2017. Influence of diet on the gut microbiome and implications for human health. J Transl Med 15:73. doi:10.1186/s12967-017-1175-ydoi: 28388917 PMC5385025

[138] Lourida I , Hannon E , Littlejohns TJ , Langa KM , Hyppönen E , Kuzma E , Llewellyn DJ . 2019. Association of lifestyle and genetic risk with incidence of dementia. JAMA 322:430–437. doi:10.1001/jama.2019.9879doi: 31302669 PMC6628594

[139] Murray CJL , Ikuta KS , Sharara F , Swetschinski L , Robles Aguilar G , Gray A , Han C , Bisignano C , Rao P , Wool E , Johnson SC , Browne AJ , Chipeta MG , Fell F , Hackett S , Haines-Woodhouse G , Kashef Hamadani BH , Kumaran EAP , McManigal B , Achalapong S , Agarwal R , Akech S , Albertson S , Amuasi J , Andrews J , Aravkin A , Ashley E , Babin F-X , Bailey F , Baker S , Basnyat B , Bekker A , Bender R , Berkley JA , Bethou A , Bielicki J , Boonkasidecha S , Bukosia J , Carvalheiro C , Castañeda-Orjuela C , Chansamouth V , Chaurasia S , Chiurchiù S , Chowdhury F , Clotaire Donatien R , Cook AJ , Cooper B , Cressey TR , Criollo-Mora E , Cunningham M , Darboe S , Day NPJ , De Luca M , Dokova K , Dramowski A , Dunachie SJ , Duong Bich T , Eckmanns T , Eibach D , Emami A , Feasey N , Fisher-Pearson N , Forrest K , Garcia C , Garrett D , Gastmeier P , Giref AZ , Greer RC , Gupta V , Haller S , Haselbeck A , Hay SI , Holm M , Hopkins S , Hsia Y , Iregbu KC , Jacobs J , Jarovsky D , Javanmardi F , Jenney AWJ , Khorana M , Khusuwan S , Kissoon N , Kobeissi E , Kostyanev T , Krapp F , Krumkamp R , Kumar A , Kyu HH , Lim C , Lim K , Limmathurotsakul D , Loftus MJ , Lunn M , Ma J , Manoharan A , Marks F , May J , Mayxay M , Mturi N , Munera-Huertas T , Musicha P , Musila LA , Mussi-Pinhata MM , Naidu RN , Nakamura T , Nanavati R , Nangia S , Newton P , Ngoun C , Novotney A , Nwakanma D , Obiero CW , Ochoa TJ , Olivas-Martinez A , Olliaro P , Ooko E , Ortiz-Brizuela E , Ounchanum P , Pak GD , Paredes JL , Peleg AY , Perrone C , Phe T , Phommasone K , Plakkal N , Ponce-de-Leon A , Raad M , Ramdin T , Rattanavong S , Riddell A , Roberts T , Robotham JV , Roca A , Rosenthal VD , Rudd KE , Russell N , Sader HS , Saengchan W , Schnall J , Scott JAG , Seekaew S , Sharland M , Shivamallappa M , Sifuentes-Osornio J , Simpson AJ , Steenkeste N , Stewardson AJ , Stoeva T , Tasak N , Thaiprakong A , Thwaites G , Tigoi C , Turner C , Turner P , van Doorn HR , Velaphi S , Vongpradith A , Vongsouvath M , Vu H , Walsh T , Walson JL , Waner S , Wangrangsimakul T , Wannapinij P , Wozniak T , Young Sharma TEMW , Yu KC , Zheng P , Sartorius B , Lopez AD , Stergachis A , Moore C , Dolecek C , Naghavi M . 2022. Global burden of bacterial antimicrobial resistance in 2019: a systematic analysis. Lancet 399:629–655. doi:10.1016/S0140-6736(21)02724-0 35065702 PMC8841637

[140] O’Neill J . 2014. Review on antimicrobial resistance: tackling a crisis for the health and wealth of nations. Review on Antimicrobial Resistance, London.

[141] Anthony WE , Wang B , Sukhum KV , D’Souza AW , Hink T , Cass C , Seiler S , Reske KA , Coon C , Dubberke ER , Burnham C-AD , Dantas G , Kwon JH . 2022. Acute and persistent effects of commonly used antibiotics on the gut microbiome and resistome in healthy adults. Cell Rep 39:110649. doi:10.1016/j.celrep.2022.110649doi: 35417701 PMC9066705

[142] Ghuneim L-AJ , Raghuvanshi R , Neugebauer KA , Guzior DV , Christian MH , Schena B , Feiner JM , Castillo-Bahena A , Mielke J , McClelland M , Conrad D , Klapper I , Zhang T , Quinn RA . 2022. Complex and unexpected outcomes of antibiotic therapy against a polymicrobial infection. ISME J 16:2065–2075. doi:10.1038/s41396-022-01252-5doi: 35597889 PMC9381758

[143] Garrett WS , Gallini CA , Yatsunenko T , Michaud M , DuBois A , Delaney ML , Punit S , Karlsson M , Bry L , Glickman JN , Gordon JI , Onderdonk AB , Glimcher LH . 2010. Enterobacteriaceae act in concert with the gut microbiota to induce spontaneous and maternally transmitted colitis. Cell Host Microbe 8:292–300. doi:10.1016/j.chom.2010.08.004doi: 20833380 PMC2952357

[144] Haran JP , Bhattarai SK , Foley SE , Dutta P , Ward DV , Bucci V , McCormick BA . 2019. Alzheimer’s disease microbiome is associated with dysregulation of the anti-inflammatory P-glycoprotein pathway. mBio 10:e00632-19. doi:10.1128/mBio.00632-19doi: 31064831 PMC6509190

[145] David A S , Taruna I , Andrew S San A , Elfi W , Masoud J A . 2018. Amyotrophic lateral sclerosis (ALS) linked to intestinal microbiota dysbiosis & systemic microbial infection in human patients: a cross-sectional clinical study. Int J Neurodegener Dis 1. doi:10.23937/ijnd-2017/1710003

[146] Boucher HW , Talbot GH , Bradley JS , Edwards JE , Gilbert D , Rice LB , Scheld M , Spellberg B , Bartlett J . 2009. Bad bugs, no drugs: no ESKAPE! an update from the infectious diseases society of America. Clin Infect Dis 48:1–12. doi:10.1086/595011doi: 19035777

[147] Stickler DJ . 2009. Proteus mirabilis biofilm formation and catheter design, p 157–190. In Denstedt J , A Atala (ed), Biomaterials and tissue engineering in urology. Woodhead Publishing.

[148] Su C-M , Kung C-T , Chen F-C , Cheng H-H , Hsiao S-Y , Lai Y-R , Huang C-C , Tsai N-W , Lu C-H . 2018. Manifestations and outcomes of patients with Parkinson’s disease and serious infection in the emergency department. Biomed Res Int 2018:6014896. doi:10.1155/2018/6014896doi: 30417011 PMC6207881

[149] Choi JG , Kim N , Ju IG , Eo H , Lim S-M , Jang S-E , Kim D-H , Oh MS . 2018. Oral administration of Proteus mirabilis damages dopaminergic neurons and motor functions in mice. Sci Rep 8:1275. doi:10.1038/s41598-018-19646-xdoi: 29352191 PMC5775305

[150] Lee DW , Ryu Y-K , Chang D-H , Park H-Y , Go J , Maeng S-Y , Hwang DY , Kim B-C , Lee C-H , Kim K-S . 2022. Agathobaculum butyriciproducens shows neuroprotective effects in a 6-OHDA-induced mouse model of Parkinson’s disease. J Microbiol Biotechnol 32:1168–1177. doi:10.4014/jmb.2205.05032doi: 36168204 PMC9628974

[151] Go J , Chang D-H , Ryu Y-K , Park H-Y , Lee I-B , Noh J-R , Hwang DY , Kim B-C , Kim K-S , Lee C-H . 2021. Human gut microbiota Agathobaculum butyriciproducens improves cognitive impairment in LPS-induced and APP/PS1 mouse models of Alzheimer’s disease. Nutr Res 86:96–108. doi:10.1016/j.nutres.2020.12.010doi: 33551257

[152] Ou Z , Deng L , Lu Z , Wu F , Liu W , Huang D , Peng Y . 2020. Protective effects of Akkermansia muciniphila on cognitive deficits and amyloid pathology in a mouse model of Alzheimer’s disease. Nutr Diabetes 10:12. doi:10.1038/s41387-020-0115-8doi: 32321934 PMC7176648

[153] He X , Yan C , Zhao S , Zhao Y , Huang R , Li Y . 2022. The preventive effects of probiotic Akkermansia muciniphila on D-galactose/AlCl3 mediated Alzheimer’s disease-like rats. Exp Gerontol 170:111959. doi:10.1016/j.exger.2022.111959doi: 36152776

[154] Goya ME , Xue F , Sampedro-Torres-Quevedo C , Arnaouteli S , Riquelme-Dominguez L , Romanowski A , Brydon J , Ball KL , Stanley-Wall NR , Doitsidou M . 2020. Probiotic Bacillus subtilis protects against α-synuclein aggregation in C. elegans. Cell Rep 30:367–380. doi:10.1016/j.celrep.2019.12.078doi: 31940482 PMC6963774

[155] Cogliati S , Clementi V , Francisco M , Crespo C , Argañaraz F , Grau R . 2020. Bacillus subtilis delays neurodegeneration and behavioral impairment in the Alzheimer’s disease model Caenorhabditis elegans. J Alzheimers Dis 73:1035–1052. doi:10.3233/JAD-190837doi: 31884470

[156] Kim H , Kim S , Park S-J , Park G , Shin H , Park MS , Kim J . 2021. Administration of Bifidobacterium bifidum BGN4 and Bifidobacterium longum BORI improves cognitive and memory function in the mouse model of Alzheimer’s disease. Front Aging Neurosci 13:709091. doi:10.3389/fnagi.2021.709091doi: 34421576 PMC8378450

[157] Kobayashi Y , Sugahara H , Shimada K , Mitsuyama E , Kuhara T , Yasuoka A , Kondo T , Abe K , Xiao J-Z . 2017. Therapeutic potential of Bifidobacterium breve strain A1 for preventing cognitive impairment in Alzheimer’s disease. Sci Rep 7:13510. doi:10.1038/s41598-017-13368-2doi: 29044140 PMC5647431

[158] Mehrabadi S , Sadr SS . 2020. Assessment of probiotics mixture on memory function, inflammation markers, and oxidative stress in an Alzheimer’s disease model of rats. Iran Biomed J 24:220–228. doi:10.29252/ibj.24.4.220doi: 32306720 PMC7275815

[159] Lee H-J , Lee K-E , Kim J-K , Kim D-H . 2019. Suppression of gut dysbiosis by Bifidobacterium longum alleviates cognitive decline in 5XFAD transgenic and aged mice. Sci Rep 9:11814. doi:10.1038/s41598-019-48342-7doi: 31413350 PMC6694197

[160] Little CS , Hammond CJ , MacIntyre A , Balin BJ , Appelt DM . 2004. Chlamydia pneumoniae induces Alzheimer-like amyloid plaques in brains of BALB/c mice. Neurobiol Aging 25:419–429. doi:10.1016/S0197-4580(03)00127-1doi: 15013562

[161] Sun J , Xu J , Yang B , Chen K , Kong Y , Fang N , Gong T , Wang F , Ling Z , Liu J . 2020. Effect of Clostridium butyricum against microglia-mediated neuroinflammation in Alzheimer’s disease via regulating gut microbiota and metabolites butyrate. Mol Nutr Food Res 64:e1900636. doi:10.1002/mnfr.201900636doi: 31835282

[162] Mohri-Shiomi A , Garsin DA . 2008. Insulin signaling and the heat shock response modulate protein homeostasis in the Caenorhabditis elegans intestine during infection *. J Biol Chem 283:194–201. doi:10.1074/jbc.M707956200doi: 17951251

[163] Wu S-C , Cao Z-S , Chang K-M , Juang J-L . 2017. Intestinal microbial dysbiosis aggravates the progression of Alzheimer’s disease in Drosophila. Nat Commun 8:24. doi:10.1038/s41467-017-00040-6doi: 28634323 PMC5478647

[164] Chongtham A , Yoo JH , Chin TM , Akingbesote ND , Huda A , Marsh JL , Khoshnan A . 2022. Gut bacteria regulate the pathogenesis of Huntington’s disease in Drosophila model. Front Neurosci 16:902205. doi:10.3389/fnins.2022.902205doi: 35757549 PMC9215115

[165] Schütze S , Döpke A , Kellert B , Seele J , Ballüer M , Bunkowski S , Kreutzfeldt M , Brück W , Nau R . 2022. Intracerebral infection with E. coli impairs spatial learning and induces necrosis of hippocampal neurons in the Tg2576 mouse model of Alzheimer’s disease. J Alzheimers Dis Rep 6:101–114. doi:10.3233/ADR-210049doi: 35530117 PMC9028720

[166] Tan FHP , Liu G , Lau S-YA , Jaafar MH , Park Y-H , Azzam G , Li Y , Liong M-T . 2020. Lactobacillus probiotics improved the gut microbiota profile of a Drosophila melanogaster Alzheimer’s disease model and alleviated neurodegeneration in the eye. Benef Microbes 11:79–89. doi:10.3920/BM2019.0086doi: 32066253

[167] Liu G , Tan FH-P , Lau S-YA , Jaafar MH , Chung FY-L , Azzam G , Liong M-T , Li Y . 2022. Lactic acid bacteria feeding reversed the malformed eye structures and ameliorated gut microbiota profiles of Drosophila melanogaster Alzheimer’s disease model. J Appl Microbiol 132:3155–3167. doi:10.1111/jam.14773doi: 32640111

[168] Ishida N , Ishihara Y , Ishida K , Tada H , Funaki-Kato Y , Hagiwara M , Ferdous T , Abdullah M , Mitani A , Michikawa M , Matsushita K . 2017. Periodontitis induced by bacterial infection exacerbates features of Alzheimer’s disease in transgenic mice. NPJ Aging Mech Dis 3:15. doi:10.1038/s41514-017-0015-xdoi: 29134111 PMC5673943

[169] Aravindraja C , Sakthivel R , Liu X , Goodwin M , Veena P , Godovikova V , Fenno JC , Levites Y , Golde TE , Kesavalu L . 2022. Intracerebral but not peripheral infection of live Porphyromonas gingivalis exacerbates Alzheimer&rsquo;s disease like amyloid pathology in APP-TgCRND8 mice. Int J Mol Sci 23:3328. doi:10.3390/ijms23063328doi: 35328748 PMC8954230

[170] Díaz-Zúñiga J , More J , Melgar-Rodríguez S , Jiménez-Unión M , Villalobos-Orchard F , Muñoz-Manríquez C , Monasterio G , Valdés JL , Vernal R , Paula-Lima A . 2020. Alzheimer’s disease-like pathology triggered by Porphyromonas gingivalis in wild type rats is serotype dependent. Front Immunol 11:588036. doi:10.3389/fimmu.2020.588036doi: 33240277 PMC7680957

[171] Balczon R , Lin MT , Lee JY , Abbasi A , Renema P , Voth SB , Zhou C , Koloteva A , Michael Francis C , Sodha NR , Pittet J-F , Wagener BM , Bell J , Choi C-S , Ventetuolo CE , Stevens T . 2021. Pneumonia initiates a tauopathy. FASEB J 35:e21807. doi:10.1096/fj.202100718Rdoi: 34384141 PMC8443149

[172] Kumar DKV , Choi SH , Washicosky KJ , Eimer WA , Tucker S , Ghofrani J , Lefkowitz A , McColl G , Goldstein LE , Tanzi RE , Moir RD . 2016. Amyloid-β peptide protects against microbial infection in mouse and worm models of Alzheimer’s disease. Sci Transl Med 8:340ra72. doi:10.1126/scitranslmed.aaf1059doi: PMC5505565 27225182

[173] Toubes E , Singh K , Yin D , Lyu R , Glick N , Russell L , Mohapatra S , Saghal N , Weinstein RA , Trenholme G . 2003. Risk factors for antibiotic-resistant infection and treatment outcomes among hospitalized patients transferred from long-term care facilities: does antimicrobial choice make a difference?. Clin Infect Dis 36:724–730. doi:10.1086/368081doi: 12627356

[174] Mitchell SL , Teno JM , Miller SC , Mor V . 2005. A national study of the location of death for older persons with dementia. J Am Geriatr Soc 53:299–305. doi:10.1111/j.1532-5415.2005.53118.xdoi: 15673356

[175] Chen J-H , Lamberg JL , Chen Y-C , Kiely DK , Page JH , Person CJ , Mitchell SL . 2006. Occurrence and treatment of suspected pneumonia in long-term care residents dying with advanced dementia. J Am Geriatr Soc 54:290–295. doi:10.1111/j.1532-5415.2005.00524.xdoi: 16460381

[176] Pop-Vicas A , Mitchell SL , Kandel R , Schreiber R , D’Agata EMC . 2008. Multidrug-resistant gram-negative bacteria in a long-term care facility: prevalence and risk factors. J Am Geriatr Soc 56:1276–1280. doi:10.1111/j.1532-5415.2008.01787.xdoi: 18557965

[177] Rowan-Nash AD , Araos R , D’Agata EMC , Belenky P . 2020. Antimicrobial resistance gene prevalence in a population of patients with advanced dementia is related to specific pathobionts. iScience 23:100905. doi:10.1016/j.isci.2020.100905doi: 32106056 PMC7044522

[178] Li W , Wu X , Hu X , Wang T , Liang S , Duan Y , Jin F , Qin B . 2017. Structural changes of gut microbiota in Parkinson’s disease and its correlation with clinical features. Sci China Life Sci 60:1223–1233. doi:10.1007/s11427-016-9001-4doi: 28536926

[179] Zhao Y , Jaber V , Lukiw WJ . 2017. Secretory products of the human GI tract microbiome and their potential impact on Alzheimer’s disease (AD): detection of lipopolysaccharide (LPS) in AD hippocampus. Front Cell Infect Microbiol 7:318. doi:10.3389/fcimb.2017.00318doi: 28744452 PMC5504724

[180] Griffen AL , Becker MR , Lyons SR , Moeschberger ML , Leys EJ . 1998. Prevalence of Porphyromonas gingivalis and periodontal health status. J Clin Microbiol 36:3239–3242. doi:10.1128/JCM.36.11.3239-3242.1998doi: 9774572 PMC105308

[181] Dominy SS , Lynch C , Ermini F , Benedyk M , Marczyk A , Konradi A , Nguyen M , Haditsch U , Raha D , Griffin C , Holsinger LJ , Arastu-Kapur S , Kaba S , Lee A , Ryder MI , Potempa B , Mydel P , Hellvard A , Adamowicz K , Hasturk H , Walker GD , Reynolds EC , Faull RLM , Curtis MA , Dragunow M , Potempa J . 2019. Porphyromonas gingivalis in Alzheimer’s disease brains: evidence for disease causation and treatment with small-molecule inhibitors. Sci Adv 5:eaau3333. doi:10.1126/sciadv.aau3333doi: 30746447 PMC6357742

[182] Flemmig TF , Milián E , Karch H , Klaiber B . 1998. Differential clinical treatment outcome after systemic metronidazole and amoxicillin in patients harboring actinobacillus actinomycetemcomitans and/or Porphyromonas gingivalis. J Clin Periodontol 25:380–387. doi:10.1111/j.1600-051x.1998.tb02459.xdoi: 9650874

[183] Shima K , Kuhlenbäumer G , Rupp J . 2010. Chlamydia pneumoniae infection and Alzheimer’s disease: a connection to remember?. Med Microbiol Immunol 199:283–289. doi:10.1007/s00430-010-0162-1doi: 20445987

[184] Balin BJ , Gérard HC , Arking EJ , Appelt DM , Branigan PJ , Abrams JT , Whittum-Hudson JA , Hudson AP . 1998. Identification and localization of Chlamydia pneumoniae in the Alzheimer’s brain. Med Microbiol Immunol 187:23–42. doi:10.1007/s004300050071doi: 9749980

[185] Kuo CC , Jackson LA , Campbell LA , Grayston JT . 1995. Chlamydia pneumoniae (TWAR). Clin Microbiol Rev 8:451–461. doi:10.1128/CMR.8.4.451doi: 8665464 PMC172870

[186] Kutlin A , Kohlhoff S , Roblin P , Hammerschlag MR , Riska P . 2005. Emergence of resistance to rifampin and rifalazil in Chlamydophila pneumoniae and Chlamydia trachomatis. Antimicrob Agents Chemother 49:903–907. doi:10.1128/AAC.49.3.903-907.2005doi: 15728882 PMC549234

[187] Hoppe T , Cohen E . 2020. Organismal protein homeostasis mechanisms. Genetics 215:889–901. doi:10.1534/genetics.120.301283doi: 32759342 PMC7404231

[188] Brignull HR , Moore FE , Tang SJ , Morimoto RI . 2006. Polyglutamine proteins at the pathogenic threshold display neuron-specific aggregation in a pan-neuronal Caenorhabditis elegans model. J Neurosci 26:7597–7606. doi:10.1523/JNEUROSCI.0990-06.2006doi: 16855087 PMC6674286

[189] Morley JF , Brignull HR , Weyers JJ , Morimoto RI . 2002. The threshold for polyglutamine-expansion protein aggregation and cellular toxicity is dynamic and influenced by aging in Caenorhabditis elegans. Proc Natl Acad Sci U S A 99:10417–10422. doi:10.1073/pnas.152161099doi: 12122205 PMC124929

[190] Vaziriyan-Sani AS , Handy RD , Walker AC , Pagolu CN , Enslow SM , Czyż DM . 2021. Automating aggregate quantification in Caenorhabditis elegans. J Vis Exp 176. doi:10.3791/62997doi: 34723951

[191] Ochoa CD , Alexeyev M , Pastukh V , Balczon R , Stevens T . 2012. Pseudomonas aeruginosa exotoxin Y is a promiscuous cyclase that increases endothelial tau phosphorylation and permeability. J Biol Chem 287:25407–25418. doi:10.1074/jbc.M111.301440doi: 22637478 PMC3408204

[192] Balczon R , Morrow KA , Zhou C , Edmonds B , Alexeyev M , Pittet J-F , Wagener BM , Moser SA , Leavesley S , Zha X , Frank DW , Stevens T . 2017. Pseudomonas aeruginosa infection liberates transmissible, cytotoxic prion amyloids. FASEB J 31:2785–2796. doi:10.1096/fj.201601042RRdoi: 28314768 PMC5471513

[193] Choi C-S , Gwin M , Voth S , Kolb C , Zhou C , Nelson AR , deWeever A , Koloteva A , Annamdevula NS , Murphy JM , Wagener BM , Pittet J-F , Lim S-TS , Balczon R , Stevens T , Lin MT . 2022. Cytotoxic tau released from lung microvascular endothelial cells upon infection with Pseudomonas aeruginosa promotes neuronal tauopathy. J Biol Chem 298:101482. doi:10.1016/j.jbc.2021.101482doi: 34896150 PMC8718960

[194] Morrow KA , Ochoa CD , Balczon R , Zhou C , Cauthen L , Alexeyev M , Schmalzer KM , Frank DW , Stevens T . 2016. Pseudomonas aeruginosa exoenzymes U and Y induce a transmissible endothelial proteinopathy. Am J Physiol Lung Cell Mol Physiol 310:L337–L353. doi:10.1152/ajplung.00103.2015doi: 26637633 PMC4754902

[195] Archambaud C , Derré-Bobillot A , Lapaque N , Rigottier-Gois L , Serror P . 2019. Intestinal translocation of enterococci requires a threshold level of enterococcal overgrowth in the lumen. Sci Rep 9:8926. doi:10.1038/s41598-019-45441-3doi: 31222056 PMC6586816

[196] Camilleri M . 2019. Leaky gut: measurement and clinical implications in humans. Gut 68:1516–1526. doi:10.1136/gutjnl-2019-318427doi: 31076401 PMC6790068

[197] Vogt NM , Kerby RL , Dill-McFarland KA , Harding SJ , Merluzzi AP , Johnson SC , Carlsson CM , Asthana S , Zetterberg H , Blennow K , Bendlin BB , Rey FE . 2017. Gut microbiome alterations in Alzheimer’s disease. Sci Rep 7:13537. doi:10.1038/s41598-017-13601-ydoi: 29051531 PMC5648830

[198] Fijan S . 2014. Microorganisms with claimed probiotic properties: an overview of recent literature. Int J Environ Res Public Health 11:4745–4767. doi:10.3390/ijerph110504745doi: 24859749 PMC4053917

[199] Li Z , Zhu H , Zhang L , Qin C . 2018. The intestinal microbiome and Alzheimer’s disease: a review. Animal Model Exp Med 1:180–188. doi:10.1002/ame2.12033doi: 30891563 PMC6388077

[200] Shamsipour S , Sharifi G , Taghian F . 2021. An 8-week administration of Bifidobacterium bifidum and Lactobacillus plantarum combined with exercise training alleviates neurotoxicity of Aβ and spatial learning via acetylcholine in Alzheimer rat model. J Mol Neurosci 71:1495–1505. doi:10.1007/s12031-021-01812-ydoi: 33715084

[201] Wang J , Ji H , Wang S , Liu H , Zhang W , Zhang D , Wang Y . 2018. Probiotic Lactobacillus plantarum promotes intestinal barrier function by strengthening the epithelium and modulating gut microbiota. Front Microbiol 9:1953. doi:10.3389/fmicb.2018.01953doi: 30197632 PMC6117384

[202] Botta C , Acquadro A , Greppi A , Barchi L , Bertolino M , Cocolin L , Rantsiou K . 2017. Genomic assessment in Lactobacillus plantarum links the butyrogenic pathway with glutamine metabolism. Sci Rep 7:15975. doi:10.1038/s41598-017-16186-8doi: 29162929 PMC5698307

[203] Zhang Y-G , Wu S , Yi J , Xia Y , Jin D , Zhou J , Sun J . 2017. Target intestinal microbiota to alleviate disease progression in amyotrophic lateral sclerosis. Clin Ther 39:322–336. doi:10.1016/j.clinthera.2016.12.014doi: 28129947 PMC5344195

[204] Jacquier V , Nelson A , Jlali M , Rhayat L , Brinch KS , Devillard E . 2019. Bacillus subtilis 29784 induces a shift in broiler gut microbiome toward butyrate-producing bacteria and improves intestinal histomorphology and animal performance. Poult Sci 98:2548–2554. doi:10.3382/ps/pey602doi: 30668816

[205] Donato V , Ayala FR , Cogliati S , Bauman C , Costa JG , Leñini C , Grau R . 2017. Bacillus subtilis biofilm extends Caenorhabditis elegans longevity through downregulation of the insulin-like signalling pathway. Nat Commun 8:14332. doi:10.1038/ncomms14332doi: 28134244 PMC5290332

[206] Jang CH , Oh J , Lim JS , Kim HJ , Kim J-S . 2021. Fermented soy products: beneficial potential in neurodegenerative diseases. Foods 10:636. doi:10.3390/foods10030636doi: 33803607 PMC8003083

[207] Hsu R-L , Lee K-T , Wang J-H , Lee LY-L , Chen RP-Y . 2009. Amyloid-degrading ability of nattokinase from Bacillus subtilis natto. J Agric Food Chem 57:503–508. doi:10.1021/jf803072rdoi: 19117402

[208] Ouyang J , Lin J , Isnard S , Fombuena B , Peng X , Marette A , Routy B , Messaoudene M , Chen Y , Routy J-P . 2020. The bacterium Akkermansia muciniphila: a sentinel for gut permeability and its relevance to HIV-related inflammation. Front Immunol 11:645. doi:10.3389/fimmu.2020.00645doi: 32328074 PMC7160922

[209] Zhang T , Li Q , Cheng L , Buch H , Zhang F . 2019. Akkermansia muciniphila is a promising probiotic. Microb Biotechnol 12:1109–1125. doi:10.1111/1751-7915.13410doi: 31006995 PMC6801136

[210] Bullich C , Keshavarzian A , Garssen J , Kraneveld A , Perez-Pardo P . 2019. Gut vibes in Parkinson’s disease: the microbiota-gut-brain axis. Mov Disord Clin Pract 6:639–651. doi:10.1002/mdc3.12840doi: 31745471 PMC6856467

[211] Asnicar F , Berry SE , Valdes AM , Nguyen LH , Piccinno G , Drew DA , Leeming E , Gibson R , Le Roy C , Khatib HA , Francis L , Mazidi M , Mompeo O , Valles-Colomer M , Tett A , Beghini F , Dubois L , Bazzani D , Thomas AM , Mirzayi C , Khleborodova A , Oh S , Hine R , Bonnett C , Capdevila J , Danzanvilliers S , Giordano F , Geistlinger L , Waldron L , Davies R , Hadjigeorgiou G , Wolf J , Ordovás JM , Gardner C , Franks PW , Chan AT , Huttenhower C , Spector TD , Segata N . 2021. Microbiome connections with host metabolism and habitual diet from 1,098 deeply phenotyped individuals. Nat Med 27:321–332. doi:10.1038/s41591-020-01183-8doi: 33432175 PMC8353542

[212] Liu J , Sun J , Wang F , Yu X , Ling Z , Li H , Zhang H , Jin J , Chen W , Pang M , Yu J , He Y , Xu J . 2015. Neuroprotective effects of Clostridium butyricum against vascular dementia in mice via metabolic butyrate. Biomed Res Int 2015:412946. doi:10.1155/2015/412946doi: 26523278 PMC4615854

[213] Zhu X , Li B , Lou P , Dai T , Chen Y , Zhuge A , Yuan Y , Li L . 2021. The relationship between the gut microbiome and neurodegenerative diseases. Neurosci Bull 37:1510–1522. doi:10.1007/s12264-021-00730-8doi: 34216356 PMC8490573

[214] Sonninen T-M , Goldsteins G , Laham-Karam N , Koistinaho J , Lehtonen Š . 2020. Proteostasis disturbances and inflammation in neurodegenerative diseases. Cells 9:2183. doi:10.3390/cells9102183doi: 32998318 PMC7601929

[215] Korovila I , Hugo M , Castro JP , Weber D , Höhn A , Grune T , Jung T . 2017. Proteostasis, oxidative stress and aging. Redox Biol 13:550–567. doi:10.1016/j.redox.2017.07.008doi: 28763764 PMC5536880

[216] Weidinger A , Kozlov AV . 2015. Biological activities of reactive oxygen and nitrogen species: oxidative stress versus signal transduction. Biomolecules 5:472–484. doi:10.3390/biom5020472doi: 25884116 PMC4496681

[217] Bomberger JM , Ye S , Maceachran DP , Koeppen K , Barnaby RL , O’Toole GA , Stanton BA . 2011. A Pseudomonas aeruginosa toxin that hijacks the host ubiquitin proteolytic system. PLOS Pathog 7:e1001325. doi:10.1371/journal.ppat.1001325doi: 21455491 PMC3063759

[218] Vasquez-Rifo A , Ricci EP , Ambros V . 2020. Pseudomonas aeruginosa cleaves the decoding center of Caenorhabditis elegans ribosomes. PLOS Biol 18:e3000969. doi:10.1371/journal.pbio.3000969doi: 33259473 PMC7707567

[219] Park SR , Lee KD , Kim UK , Gil YG , Oh KS , Park BS , Kim GC . 2010. Pseudomonas aeruginosa exotoxin A reduces chemoresistance of oral squamous carcinoma cell via inhibition of heat shock proteins 70 (HSP70). Yonsei Med J 51:708–716. doi:10.3349/ymj.2010.51.5.708doi: 20635445 PMC2908850

[220] Keestra-Gounder AM , Byndloss MX , Seyffert N , Young BM , Chávez-Arroyo A , Tsai AY , Cevallos SA , Winter MG , Pham OH , Tiffany CR , de Jong MF , Kerrinnes T , Ravindran R , Luciw PA , McSorley SJ , Bäumler AJ , Tsolis RM . 2016. NOD1 and NOD2 signalling links ER stress with inflammation. Nature 532:394–397. doi:10.1038/nature17631doi: 27007849 PMC4869892

[221] Cheng L , Chen L , Wei X , Wang Y , Ren Z , Zeng S , Zhang X , Wen H , Gao C , Liu H . 2018. NOD2 promotes dopaminergic degeneration regulated by NADPH oxidase 2 in 6-hydroxydopamine model of Parkinson’s disease. J Neuroinflammation 15:243. doi:10.1186/s12974-018-1289-zdoi: 30157869 PMC6116377

[222] Saresella M , La Rosa F , Piancone F , Zoppis M , Marventano I , Calabrese E , Rainone V , Nemni R , Mancuso R , Clerici M . 2016. The NLRP3 and NLRP1 inflammasomes are activated in Alzheimer’s disease. Mol Neurodegener 11:23. doi:10.1186/s13024-016-0088-1doi: 26939933 PMC4778358

[223] Cheon SY , Kim J , Kim SY , Kim EJ , Koo B-N . 2020. Inflammasome and cognitive symptoms in human diseases: biological evidence from experimental research. Int J Mol Sci 21:1103. doi:10.3390/ijms21031103doi: 32046097 PMC7036918

